# ZNF274 Recruits the Histone Methyltransferase SETDB1 to the 3′ Ends of ZNF Genes

**DOI:** 10.1371/journal.pone.0015082

**Published:** 2010-12-08

**Authors:** Seth Frietze, Henriette O'Geen, Kimberly R. Blahnik, Victor X. Jin, Peggy J. Farnham

**Affiliations:** 1 Department of Pharmacology and the Genome Center, University of California Davis, Davis, California, United States of America; 2 Department of Biomedical Informatics, The Ohio State University, Columbus, Ohio, United States of America; Université Paris-Diderot, France

## Abstract

Only a small percentage of human transcription factors (e.g. those associated with a specific differentiation program) are expressed in a given cell type. Thus, cell fate is mainly determined by cell type-specific silencing of transcription factors that drive different cellular lineages. Several histone modifications have been associated with gene silencing, including H3K27me3 and H3K9me3. We have previously shown that genes for the two largest classes of mammalian transcription factors are marked by distinct histone modifications; homeobox genes are marked by H3K27me3 and zinc finger genes are marked by H3K9me3. Several histone methyltransferases (e.g. G9a and SETDB1) may be involved in mediating the H3K9me3 silencing mark. We have used ChIP-chip and ChIP-seq to demonstrate that SETDB1, but not G9a, is associated with regions of the genome enriched for H3K9me3. One current model is that SETDB1 is recruited to specific genomic locations via interaction with the corepressor TRIM28 (KAP1), which is in turn recruited to the genome via interaction with zinc finger transcription factors that contain a Kruppel-associated box (KRAB) domain. However, specific KRAB-ZNFs that recruit TRIM28 (KAP1) and SETDB1 to the genome have not been identified. We now show that ZNF274 (a KRAB-ZNF that contains 5 C2H2 zinc finger domains), can interact with KAP1 both in vivo and in vitro and, using ChIP-seq, we show that ZNF274 binding sites co-localize with SETDB1, KAP1, and H3K9me3 at the 3′ ends of zinc finger genes. Knockdown of ZNF274 with siRNAs reduced the levels of KAP1 and SETDB1 recruitment to the binding sites. These studies provide the first identification of a KRAB domain-containing ZNF that is involved in recruitment of the KAP1 and SETDB1 to specific regions of the human genome.

## Introduction

Transcription factors are key regulators involved in translating genomic information into cellular and organismal phenotypes. Previous studies have suggested that some transcription factors are ubiquitously expressed (such as members of the E2F family); presumably these factors regulate genes whose functions are necessary for all cell types. However, a large number of transcription factors are expressed in only a few specific tissues (e.g. the testis-specific zinc finger protein ZBTB32); presumably these factors regulate genes whose function must be limited to those specific tissues [1]. Although only a small percentage of human transcription factors have been well characterized, previous studies suggest that it is critical that transcription factors are properly controlled, being expressed only in the appropriate cell type. For example, the inappropriate expression of certain transcription factors has been clearly linked to human diseases such as cancers and neurological and developmental disorders [1]. In pluripotent embryonic stem cells many genes involved in creating specific differentiated cell types are kept at very low levels. However, once a differentiation program has been induced, then genes specific for a given cell state are turned on. Contained within these sets of differentiation-responsive genes are tissue-specific transcription factors. Our work [2], [3] and other studies [4], [5], [6] have revealed that epigenetic mechanisms (both DNA methylation and histone modifications) are responsible for silencing cell type-specific transcription factors in pluripotent cells.

Transcription factors are often classified according to their DNA binding domains, which provide useful information concerning their DNA binding patterns and their evolutionary relatedness. It is estimated that there are ∼1400 DNA binding site-specific transcription factors in human cells [1], [7], [8], [9]. However, over 80% of the site-specific transcription factors encoded in the human genome can be grouped into three categories; the C2H2 zinc finger domain factors (675 genes), homeodomain factors (257 genes), and helix–loop–helix factors (87 genes). We have previously shown that the genes belonging to the two largest groups of transcription factors are regulated by two different epigenetic marks; in gene ontology analyses, the most enriched class of transcription factor genes marked by H3K9me3 is C2H2 zinc finger transcription factors and the most enriched class of transcription factor genes marked by H3K27me3 is homeodomain transcription factors [2], [3], [10]. These results suggest that distinct epigenetic regulatory complexes must be dedicated to controlling expression of zinc finger vs. homeobox domain transcription factors. We [10], [11], [12] and others [13], [14], have shown that components of Polycomb Repressive Complex 2 (PRC2) co-localize with the H3K27me3 mark. However, the exact mechanism by which histone methylases are recruited to zinc finger transcription factor genes is not known.

Initial studies of H3K27me3 and H3K9me3 using ChIP-chip and promoter arrays identified large sets of promoters that were distinguished by these two marks, often in a cell type-specific pattern [3], [10], [14], [15]. However, when studies were expanded to ChIP-chip using genomic tiling arrays and then to genome-wide ChIP-seq, it became clear that H3K27me3 and H3K9me3 were not only found at promoter regions but that these marks could also spread over larger genomic regions. For H3K27me3, the spreading patterns are generally found over entire HOX gene clusters, including coding, intragenic, and intergenic reigons [10]. The H3K9me3 mark can also spread over large regions, such as centromeres, transposons, and tandem repeats [16], [17], [18]. In addition, we have previously shown that the 3′ exons of many zinc finger genes (ZNFs) are specifically covered by H3K9me3 [3]. Other studies in progress are focused on determining whether the 3′ exons of ZNF genes correspond to alternative promoters. However, the goal of this current study is to identify the DNA binding factor that recruits a regulatory histone methyltransferase to the 3′ ends of the C2H2 zinc finger genes. To achieve this goal, it is necessary to first identify the histone methyltransferase that colocalizes with H3K9me3 at zinc finger genes, then to identify a DNA binding factor that colocalizes with the histone methyltransferase, and then finally to demonstrate that the identified DNA binding factor is involved in recruitment of the histone methyltransferase to 3′ exons of ZNF genes. Several histone methyltransferases have been implicated in methylation of lysine 9 of histone H3, including G9a and SETDB1 [19]. We investigate the role of these two histone methyltransferases in regulating the H3K9me3 mark using genome-scale ChIP-chip and ChIP-seq. Our studies demonstrate that SETDB1, but not G9a, overlaps with H3K9me3 in K562 cells. We go on to test the model that SETDB1 is recruited to specific genomic locations via interaction with the corepressor TRIM28 (KAP1), which is in turn recruited to the genome via interaction with zinc finger transcription factors that contain a Kruppel-associated box (KRAB) domain. Finally, we identify a KRAB-ZNF transcription factor that co-localizes with H3K9me3 on C2H2 zinc finger clusters in several different cell types and show that this DNA binding factor is involved in targeting epigenetic regulatory complexes to the human genome.

## Results

### SETDB1, but not G9a, correlates with H3K9me3 genomic localization patterns

**Figure 1:**
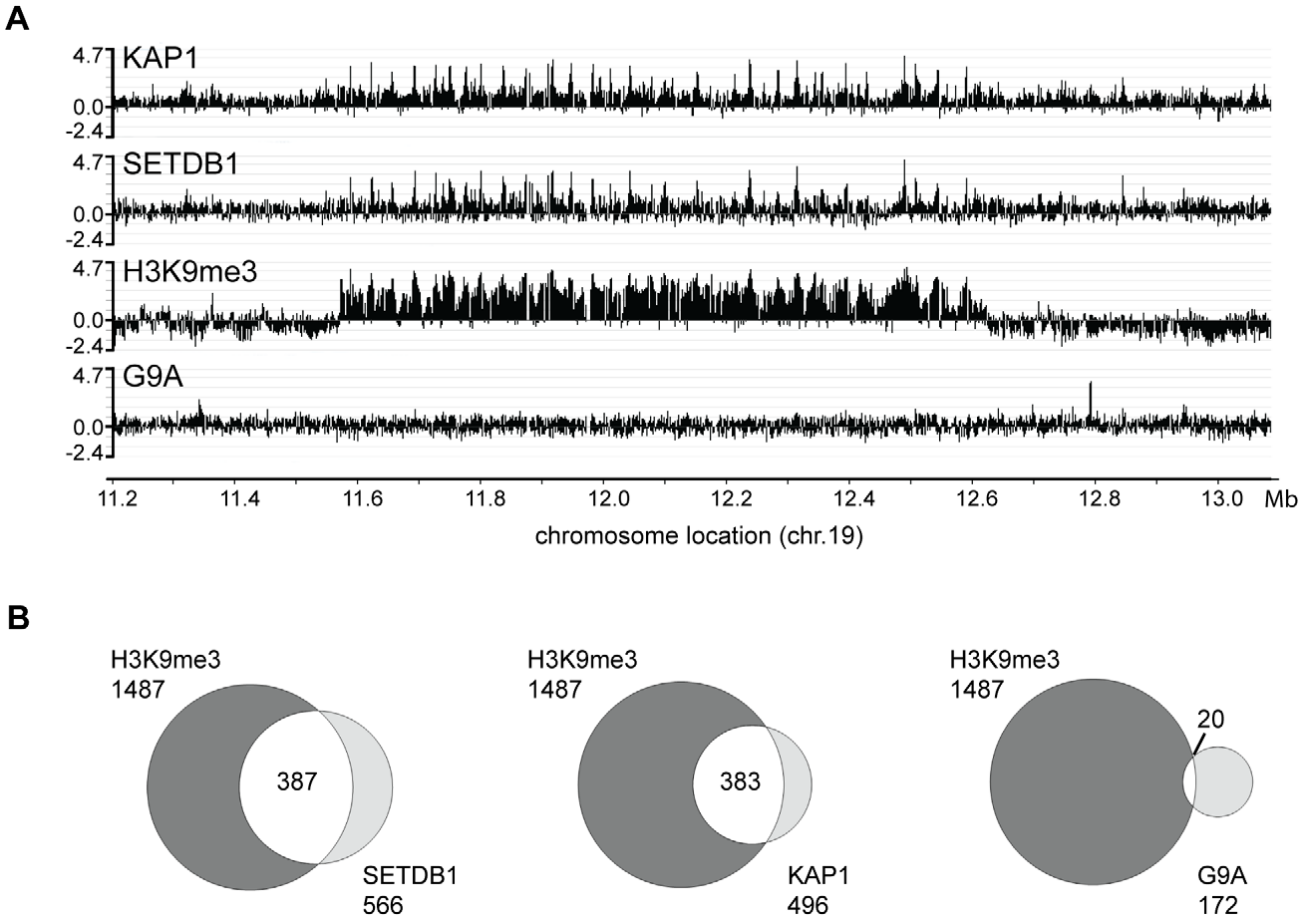
SETDB1, but not G9a, colocalizes with H3K9me3 on chromosome 19. (A) ChIP-chip binding patterns of KAP1, SETDB1, H3me3K9 and G9a are compared at a zinc finger gene cluster on chromosome 19. The log2 (ratio) values reflecting the ChIP enrichments are plotted on the y axes and chromosomal coordinates are shown on the x axis. (B) Peaks were called using the ChIP-chip array data for SETDB1, G9a, and KAP1 using the Mayacamas peak calling program. The total number of peaks for each data set are shown; the intersection indicates the number of SETDB1, KAP1, and G9a peaks that overlap with the H3K9me3 peaks.

As a first step toward understanding the mechanisms by which zinc finger transcription factor genes are marked by H3K9me3, we wished to identify the responsible histone methylase; possible candidates included G9a/KMT1C, GLP/KMT1D, SETDB1/KMT1E, Suv39h1/KMT1A and Suv39h2/KMT1B [20], [21], [22], [23], [24], [25], [26]. We used antibodies that recognize H3K9me3, G9a, or SETDB1 and performed ChIP-chip (chromatin immunoprecipitation followed by hybridization to oligonucleotide arrays) assays to compare the binding patterns of G9a and SETDB1 with the pattern of H3K9me3 (we note that we have not been able to obtain robust enrichments in ChIP assays using antibodies to other H3K9 methylases such as Suv39h1/KMT1A or Suv39h2/KMT1B). The majority of C2H2 zinc finger genes reside on chromosome 19, having expanded into large clusters of highly related genes at several evolutionary stages, including at the appearance of vertebrates and again substantially during the emergence of mammals and primates [27]. Therefore, we began our studies by performing ChIP-chip using arrays that include the entirety of chromosome 19. ChIP samples were obtained using K562 cells, amplified, and applied to the genomic tiling arrays. We identified many binding sites for the histone methyltransferase G9a (see **Supplementary Figure S1** for examples of G9a peaks). However, G9a does not bind to the same regions of the genome as are covered by the H3K9me3 mark (**Figure 1**). The peaks that were observed confirmed to be true G9a binding sites, as determined by PCR analysis of subsequent ChIP assays (see **Supplementary Figure S1**) and by the fact that they localized to sites bound by H3K9me2, not H3K9me3 (H. O'Geen, unpublished data). In contrast, the binding pattern of the histone methyltransferase SETDB1 showed a large overlap with the H3K9me3 marks (**Figure 1A**). One current model in the field is that SETDB1 is brought to the DNA via interaction with the TRIM28 (also known as KAP1) corepressor [20]. Therefore, we also performed ChIP-chip with an antibody to KAP1 and showed that both KAP1 and SETDB1 were bound to the same genomic locations. We next wished to compare the overlap of all target sites for H3K9me3 with the SETDB1 and KAP1 peaks. However, because the H3K9me3 binding pattern has many very broad regions, standard peak-calling programs are not appropriate. Therefore, we developed a modified version of our Tamalpais ChIP-chip peak calling program [28] that smoothes oligonucleotide intensities using a sliding average, and allows gaps caused by missing oligonucleotides (the result of the repetitive nature of H3K9me3 target regions); see methods for more details concerning the Mayacamas peak-calling program). Using the Mayacamas program, we identified 1487 target regions for H3K9me3, 566 target sites for SETDB1, 496 target sites for KAP1 and 172 target sites for G9a on chromosome 19. We found that most of the SETDB1 and KAP1 sites on chromosome 19 were also sites for H3K9me3, but that very few G9a sites were located in regions covered by H3K9me3 (**Figure 1B**). A list of all the ChIP-chip arrays can be found in **Supplementary Table S1** and the chromosome 19 genomic regions bound by KAP1, SETDB1, G9a, and H3K9me3 can be found in **Supplementary Table S2.**

The ChIP-chip experiments suggested a general relationship between H3K9me3 and SETDB1. However, these analyses were not genome-wide. The advent of the ChIP-seq technology now allows for a more economical and precise mapping of chromatin patterns throughout the human genome and therefore we next performed ChIP-seq experiments for H3K9me3, SETDB1, and KAP1 in K562 cells (see **Supplementary Table S1** for a summary of the ChIP-seq samples analyzed in this study). As with the ChIP-chip data, analysis of chromatin patterns from ChIP-seq data required modification of our standard peak-calling program, Sole-search [29]. Therefore, we adapted Sole-search to call broader peaks (for more details see Methods) and then identified 3866 target regions for H3K9me3, 302 sites for SETDB1, and 155 sites for KAP1 on chromosome 19. Comparison of peak sets identified using ChIP-chip and ChIP-seq for H3K9me3, SETDB1, and KAP1 and a comparison of the binding patterns for H3K9me3, SETDB1, and KAP1 using the different platforms is shown in **Supplementary Figure S6**.

### ZNF274 co-localizes with the KAP1 and SETDB1

Although it is clear that KAP1 and SETDB1 co-localize with H3K9me3, neither of these two proteins has a specific DNA binding domain and therefore additional factors are required to recruit the complex to specific regions of the genome. KAP1 contains an N-terminal protein interaction domain (the RBBC domain) that can interact in vitro with a KRAB domain [30], [31]. KRAB domains are found in a variety of different proteins [32], [33], [34], but the most prevalent set of KRAB domain containing-proteins are a subset of the family of C2H2 zinc finger transcription factors (ZNFs); approximately 300 C2H2 ZNFs contain a KRAB domain [35], [36]. Most of the previous studies of KRAB domains have been performed by artificially tethering an isolated KRAB domain to the chromatin using transiently introduced or stably integrated artificial reporter constructs [37], [38], [39]. To date there have been no studies that have shown similar cellular binding patterns of a KRAB domain containing protein and H3K9me3. In fact, the only genome-wide ChIP-seq analysis of a KRAB-ZNF showed that there was essentially no overlap between the ∼5,000 ZNF263 binding sites and the H3K9me3 chromatin pattern [40]. Therefore, it is not known which site-specific factor(s) is involved in recruiting the KAP1/SETDB1 epigenetic regulatory complex to the genome under normal physiological conditions.

In general, KRAB-ZNFs are expressed at low levels and many are tissue-specific [33]. We therefore obtained antibodies to numerous KRAB-ZNFs (more than 25 different antibodies were tested), performed western blots to identify KRAB-ZNFs that were expressed in K562 cells, and then performed immunoprecipitation experiments to determine if the antibodies for the expressed factors would potentially work in ChIP assays. Finally, we performed ChIP-seq analysis using antibodies to ZNF14, ZNF180, ZNF195, ZNF212, ZNF689, and ZNF274. Of these, the only one that showed binding to the genome was ZNF274. Therefore, we focused our analyses on this KRAB-ZNF. As shown in **Supplementary Figure S2**, we found that ZNF274 is expressed in GM12878, HepG2, HeLa, and K562 cells. ZNF274 contains 5 DNA binding zinc finger domains, a protein-protein interaction SCAN domain, and 2 KRAB domains. A published study demonstrated that ZNF274 KRAB domains could repress transcription of a synthetic promoter construct when fused to the Gal4 DNA binding domain [41]. Although the previous work did not determine if the repression mediated by a GAL4-ZNF274 KRAB domain fusion protein involved KAP1, it did suggest that perhaps ZNF274 would be a good candidate for further analyses.

**Figure 2:**
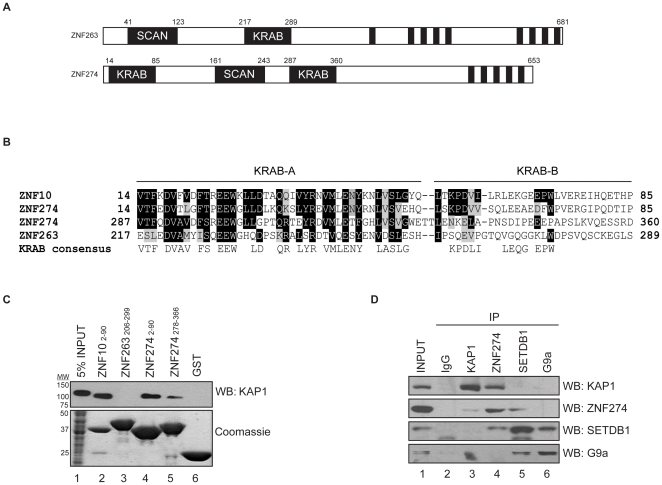
A KRAB-domain containing protein ZNF274 interacts with the KAP1 corepressor both in vitro and in vivo. A) Schematics of ZNF263 and ZNF274 proteins are shown, indicating the position of the KRAB domains (potential KAP1-interacting domains), the SCAN domains, and the zinc fingers (DNA binding domains). B) The KRAB domains of ZNF274, ZNF263, and ZNF10 are compared at the amino acid level. C) Purified KRAB-GST fusion proteins are shown in the bottom panel. In the top panel, nuclear proteins from K562 cells that bound to the indicated KRAB-GST fusion proteins were analyzed using an antibody to KAP1. D) Co-immunoprecipitation of endogeneous ZNF274 with KAP1 and SETDB1, but not with G9a. Control IgG, KAP1, ZNF274, SETDB1, and G9a antibodies were used in immunoprecipitation reactions using K562 cell extracts and were analyzed by western blotting using the indicated antibodies.

To determine whether the KRAB domains of ZNF274 could recruit KAP1, we first used an in vitro system. We used the ZNF10 KRAB domain as a positive control because the KRAB domain of this factor has been shown to interact with KAP1 in vitro [31]; unfortunately, we could not detect this protein by western blot using a series of commercially available antibodies (data not shown) and therefore we could not test if ZNF10 recruits the KAP1/SETDB1 complex in cells. We used the ZNF263 KRAB domain as a negative control, because we have previously shown that it does not co-localize with the KAP1/SETDB1 complex [40]. Portions of these two ZNFs (**Figure 2A**) corresponding to the KRAB domains were generated as recombinant GST fusion proteins and were incubated with nuclear extract from K562 cells. The association with KAP1 was analyzed by western blotting using antibodies to KAP1. As shown in **Figure 2C**, the KRAB domain of ZNF10, but not of ZNF263, can precipitate KAP1. We next tested the two KRAB domains present in ZNF274 and found that regions containing either of the KRAB domains of ZNF274 (amino acids 2-90 and 278-366) could interact with KAP1 in vitro, although the more N-terminal KRAB domain of ZNF274 appeared to have higher affinity for KAP1. A sequence alignment (**Figure 2B**) demonstrates that the first KRAB domain of ZNF274 is virtually identical to a KRAB domain consensus sequence derived from an alignment of the KRAB domains from 10 independent zinc finger gene products [30]. The KRAB domain of ZNF263, however, has substitutions in amino acids corresponding to critical residues in the KRAB domain consensus sequence, including the MLE sequence that occurs in the context of the highly conserved VMLENY motif common to many KRAB A boxes and critical for repression. This leucine residue is one of the heptad repeats of leucines (MX_6_LX_6_LX_6_L, where X is any amino acid) capable of forming an amphipathic helix [30]. The lack of the hydrophobic interaction domain may explain the inability of ZNF263 to interact with KAP1 in this assay. These biochemical experiments emphasize the need to check each KRAB-ZNF for interaction with KAP1; simply having a KRAB domain does not necessarily ensure interaction with KAP1. The second KRAB domain of ZNF274 contains the MLE sequence but lacks a leucine residue in the A box helical region as well as leucine residues in the B box, possibly explaining its weaker association with KAP1 compared to the first ZNF274 KRAB domain. To confirm the interaction between KAP1 and ZNF274 we performed co-immunoprecipitation experiments in K562 whole cell extracts using antibodies against endogenous proteins. These experiments demonstrated that endogenous ZNF274 specifically co-precipitates with KAP1 and SETDB1 but not with G9a or control IgG (**Figure 2D**). Similarly, ZNF274 co-precipitated both KAP1 and SETDB1 but not G9a (*lane 4*). Together, these data suggest that KAP1/SETDB1 associate with ZNF274 in vivo. It should be noted that we failed to detect sufficient levels of SETDB1 in KAP1 immunoprecipitation reactions, and vice versa (*lanes 3 and 5, respectively*). Also, despite significant co-precipitation of SETDB1 with G9a immunoprecipitations, we failed to detect co-precipitation of G9a with SETDB1 in these experiments (*lane 6*). Nevertheless, we conclude that ZNF274 can interact with KAP1 and SETDB1 in cells.

**Figure 3:**
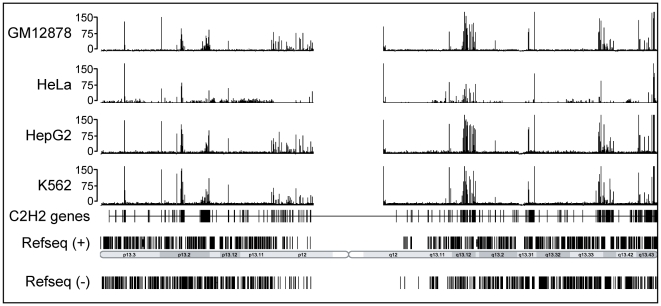
ZNF274 binds to C2H2 zinc finger genes. ChIP-seq binding patterns of ZNF274 from GM12878, HeLa, HepG2, and K562 cells are compared to the location of all C2H2 zinc finger genes on chromosome 19. The number of tags reflecting the ChIP enrichments are plotted on the y axes and chromosomal coordinates are shown on the x axis.

Having shown that ZNF274 interacts with KAP1 in vitro and in vivo, the next step was to determine if ZNF274 co-localizes with KAP1 on the genome. Because there are no known binding sites for ZNF274, we could not test the quality of the ChIP or the library before proceeding to ChIP-seq experiments. However, we did show that the ZNF274 antibody was capable of immunoprecipitating ZNF274 protein from nuclear extract (**Supplementary Figure S2B**). We performed two ChIP assays using K562 cells grown on separate days, made and sequenced libraries, and called peaks using Sole-search [29]. We obtained 7,654,486 uniquely mapping tags (i.e. tags that map to only one place in the human genome) for replicate 1 and 11,341,812 uniquely mapping tags for replicate 2. However, the number of peaks identified for each replicate was fairly small; 192 peaks for replicate 1 and 273 peaks for replicate 2. To determine the quality of the ChIP-seq data, we used the ENCODE overlap standards. Briefly, we compared the top 40% of the peaks in one replicate to the entire set of peaks (after truncating the peak lists to the same length) in the other replicate. We found a 95 and 100% overlap for the 2 comparisons, indicating that the called peaks were essentially identical in the two replicates. We then merged the uniquely mapping tags from both sequence runs and called peaks on the merged dataset to obtain a final number of 337 peaks. Because this was a very small number of binding sites (most factors that we have tested by ChIP-seq have between 1,000 and 30,000 binding sites; Frietze, O'Geen, Xu, and Farnham, unpublished data), we also performed ChIP-seq for ZNF274 in 3 other cell types. We identified 469 binding sites in HepG2 cells, 336 binding sites in GM12878 cells, and 220 binding sites in HeLa cells (see **Table 1** for a detailed characterization of all peaks called in the ChIP-seq experiments). Although the number of ZNF274 peaks was small for each cell type, the sets of peaks were similar in all cells; see **Figure 3** for a comparison of ZNF274 binding in 4 different cell types and **Supplementary Table S3** for a list of the ZNF274 peaks identified in K562, HeLa, HepG2, and GM12878 cells.

**Table 1:** Summary of ChIP-seq data.

Dataset/cell type	SETDB1/K562	KAP1/K562	H3K9me3/K562	ZNF274/K562	ZNF274/HepG2	ZNF274/GM12878	ZNF274/HeLa
Number of peaks:	3,238	3,085	16246	337	469	336	220
Average peak height:	27	30.07	18.14	49	42	43	38
Median peak height:	25	25	16	34	29	31	33
Highest peak:	102	185	141	195	195	166	117
Lowest peak:	14	16	14	17	16	15	16
Average peak width:	547	385.16	1151.44	649	551.86	604.62	469
# uniquely mapped reads	21,434,178	26,458,932	43,691,075	18,856,543	22,363,343	10,487,897	18,852,757

Numbers are from the analysis of two merged replicate datasets.

**Figure 4:**
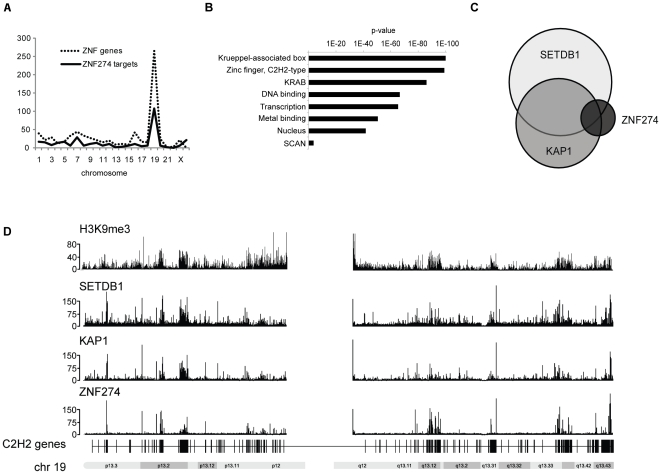
ZNF274 binds specifically to ZNF genes. A) The number of ZNF274 binding sites in K562 cells is shown for each chromosome (solid line). Also shown is the number of C2H2 ZNF genes encoded on each chromosome (dotted line). B) The ZNF274 target genes in K562 cells were analyzed using the DAVID gene ontology program. Shown are the enriched terms and P-value of enrichments of the target genes. C) Overlaps of the peaks sets of ZNF274, SETDB1, and KAP1 are shown; 85% of the 337 ZNF274 peaks, 52% of the 3237 SETDB1 peaks, and 54% of the 2027 KAP1 peaks overlap with H3K9me3. D) The ChIP-seq binding patterns of ZNF274, KAP1, SETDB1, and H3me3K9 are compared on chromosome 19. The number of tags reflecting the ChIP enrichments are plotted on the y axes and chromosomal coordinates are shown on the x axis.

**Figure 5:**
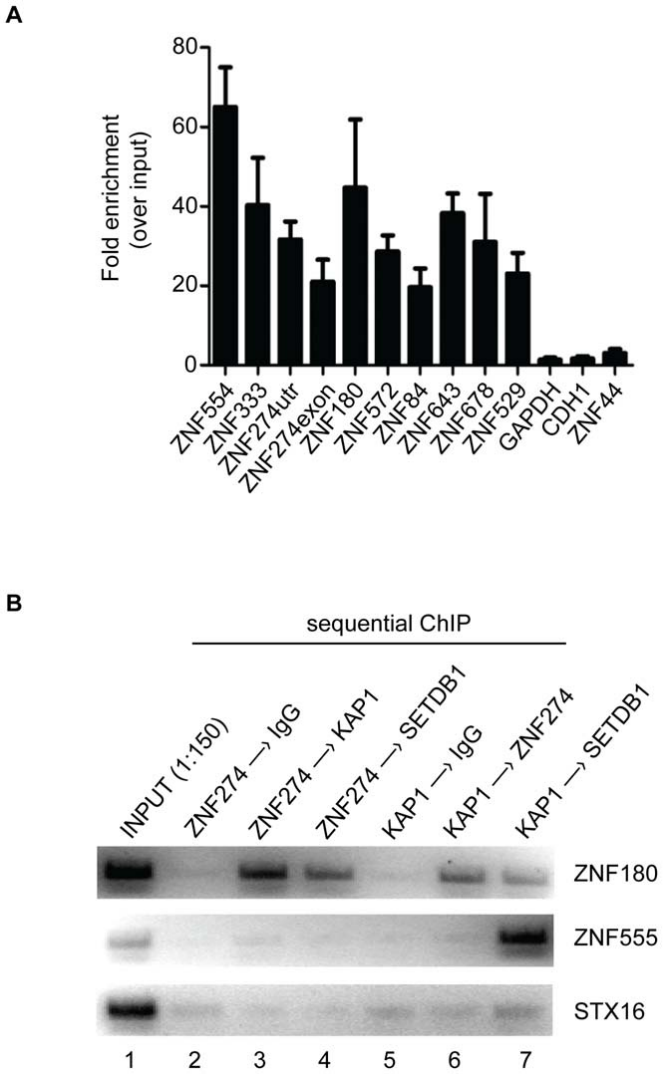
Co-occupancy of ZNF274, KAP1 and SETDB1 at a ZNF 3′ end in vivo. A) ChIP-qPCR confirmation of a set of ZNF274 targets. Quantitative real-time PCR (qPCR) for 10 target regions identified by ChIP-seq and three negative regions (GAPDH, CDH1, and ZNF44) was performed. The fold enrichment of each site was calculated as 2 to the power of the cycle threshold (cT) difference between input chromatin and ChIP samples. The results in the graph are the mean of three independent replicates with standard deviation. Primers used in these experiments can be found in **Supplementary Table S5**. B) Sequential chromatin immunoprecipitation of ZNF274 and KAP1 in K562 cells. ZNF274 or KAP1 ChIP samples were sequentially immunoprecipitated using the indicated antibodies. The samples were analyzed by PCR and agarose gel electrophoresis with ethidium bromide staining using specific primer sets to *ZNF180*, a zinc finger gene bound by ZNF274; *ZNF555,* a zinc finger gene not bound by ZNF274, but bound by KAP1; *STX16*, and a non-zinc finger gene bound by neither ZNF274 nor KAP1.

We performed follow-up analyses of the ZNF274 binding sites from K562 cells using the Sole-search location analysis tool kit [29]; see also http://chipseq.genomecenter.ucdavis.edu/cgi-bin/chipseq.cgi. As shown in **Figure 4A**, the majority of the binding sites for ZNF274 are on chromosome 19. As mentioned above, most C2H2 zinc finger genes are located on chromosome 19, suggesting that ZNF274 may in fact be involved in recruiting the SETDB1 histone methyltransferase to the family of C2H2 zinc finger genes. A comparison of the ZNF274, SETDB1, and KAP1 peak sets is shown in **Figure 4C** whereas ChIP-seq binding patterns of ZNF274, KAP1, SETDB1, and H3K9me3 throughout the entire chromosome 19 are shown in **Figure 4D** (also see **Supplementary Figure S9** for views of individual binding sites). The strong similarity in the binding patterns suggest that ZNF274 does in fact bind to C2H2 zinc finger genes; accordingly, a gene ontology analysis of the genes bound by ZNF274 shows that KRAB and SCAN domain containing zinc finger genes are essentially the only enriched category of genes (**Figure 4B**). ZNF274 binding to the 3′ ends of ZNF genes was confirmed using ChIP-qPCR (**Figure 5A**)**.**

### Co-occupancy of ZNF274, KAP1 and SETDB1 at ZNF 3′ ends in vivo

The observation that ZNF274 binds to the 3′ ends of certain ZNF gene targets also bound by KAP1 and SETDB1 in K562 cells does not indicate whether these factors are simultaneously bound at the same loci. We therefore used sequential chromatin immunoprecipitation to determine co-occupancy of ZNF274 with KAP1 or SETDB1 at the 3′ ends of ZNF genes *in vivo*. Specifically, we first performed ChIPs with antibodies for one factor (i.e. ZNF274 or KAP1), eluted the protein-DNA complexes and then immunoprecipitated the resulting sample with antibodies for the other factor (i.e. ZNF274, KAP1, SETDB1, or control IgG). We tested the eluates of sequential ChIP reactions by PCR using primers to the ZNF274 binding site *ZNF180* and to control *ZNF555* and *STX16* binding sites. Using this assay, co-occupancy of ZNF274 and KAP1 at *ZNF180* was observed when the first ChIP was performed with ZNF274 followed by KAP1 (**Figure 5B**, *lane 3*). In the reciprocal experiment in which KAP1-bound targets were immunoprecipitated first, *ZNF180* shows specific enrichment upon subsequent immunoprecipitation with ZNF274 antibodies but not with control IgG (*lanes 6 and 5, respectively*). Similarly, when ZNF274 or KAP1 ChIPs were sequentially immunoprecipitated with SETDB1 antibodies, there is specific enrichment of the *ZNF180* binding site (*lanes 4 and 7, respectively*). Conversely, the control *ZNF555* or *STX16* binding sites are not enriched in any of the ZNF274 ChIPs. The *ZNF555* binding site is specifically enriched when KAP1 ChIPs were followed by SETDB1, but not by ZNF274 or control IgG sequential immunoprecipitations (*lanes 7, 6 and 5, respectively*). Therefore, we conclude that ZNF274, KAP1 and SETDB1 co-occupy the *ZNF180* 3′ end binding site in vivo.

### Characterization of ZNF274 binding sites

**Figure 6:**
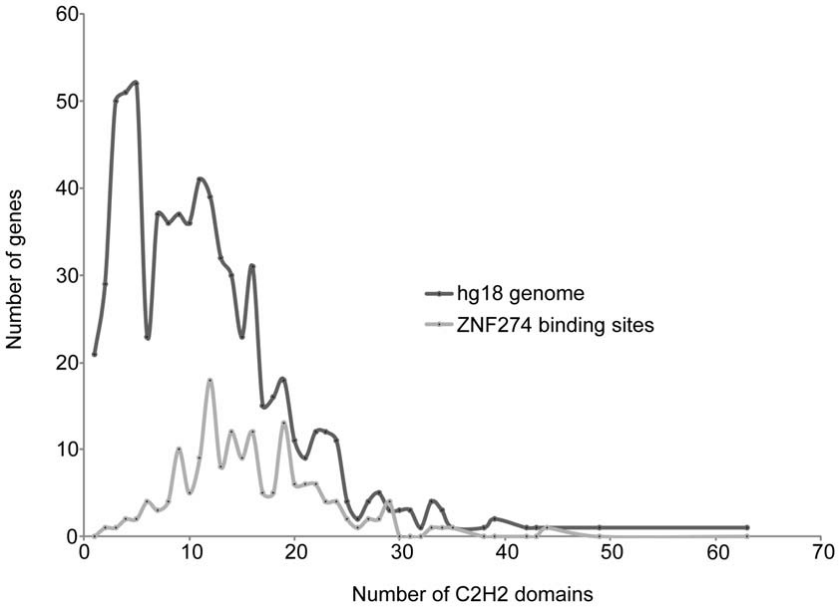
ZNF274 binds to a gene containing multiple zinc finger domains. The number of finger domains in the C2H2 genes bound by ZNF274 are shown (solid line), along with the number of finger domains found in all 712 C2H2 zinc finger genes encoded in the hg18 human genome build (dotted line).

We next performed a series of additional characterizations of the ZNF274 binding sites. First, we determined the expression level of ZNF274 target genes in K562 cells (see Methods for details). Analysis of this set of genes showed that they were expressed at low levels, in comparison to the analysis of the K562 cell transcriptome (**Supplementary** **Figure S7**). We also compared the set of C2H2 zinc finger genes that were bound by ZNF274 to the entire set of 712 C2H2 zinc finger genes encoded in the human genome. As shown in **Figure 6**, most C2H2 zinc finger genes bound by ZNF274 contain between 9 and 19 finger domains, as opposed to the overall set of C2H2 zinc finger genes which consists of a large number of genes having 1-6 finger domains and another large set of genes having 7-12 finger domains. We also note that although there are very few C2H2 genes encoded in the human genome that have more than 25 finger domains (96 of the 712 C2H2 genes), 43% of these are bound by ZNF274.

As indicated above, no binding sites had previously been identified for ZNF274 prior to our ChIP-seq studies. Therefore, we wished to determine whether a ZNF274 consensus motif could be identified from the ChIP-seq data. We analyzed 50 nt on each side of the midpoint of the 337 ZNF274 peaks from K562 cells. We chose this range because other studies have shown that a known consensus motif for a factor can be found in 75% of the peaks if ∼50 nucleotides from the center of the peak is used for analysis [42]. Motif finding for ZNF274 is complicated by the fact that the binding sites are contained with the final coding exon of the C2H2 target genes. All C2H2 target genes contain multiple zinc finger domains and conserved linker motifs between each finger. Therefore, many conserved motifs would be identified in the set of ZNF274 targets that would essentially correspond to coding sequences if standard backgrounds were used. Therefore, we used as our background a set of 3′ exons from C2H2 zinc finger genes NOT bound by ZNF274. Using our W-ChIPsmotifs program [43], [44], we identified a number of motifs that were highly enriched in the ZNF274 binding sites but not in the control set of C2H2 3′ exons (**Supplementary Table S4**). ZNF274 contains 5 zinc fingers and if each finger contacted 3 nucleotides of DNA the motif would be expected to be 15 residues in length. However, it is not known if all fingers in multi-finger DNA binding factors contact DNA. Also, we do not know if ZNF274 works as a monomer, as a homodimer, or as a heterodimer with another factor. In support of the possibility that ZNF274 may use different sets of fingers to bind to different motifs, we identified several different motifs that were highly enriched in the ZNF274 binding sites. A list of the entire set of enriched motifs along with the position weight matrices is shown in **Supplementary Figure S4**, and similarity of each enriched motif to the closest known motif for other factors is shown in **Supplementary Figure S5**. We note the ZNF274 motifs do not have high similarity to binding sites of other factors, but we did identify several quite long motifs. As shown in **Figure S8**, we found 210 copies of the M1 motif and 199 copies of the M11 motif in the set of 337 ZNF274 K562 peaks. We searched the entire human genome using the M11 and M1 PWMs and found that at the highest stringency (1.00/0.99), there are only 724 matches to the M1 PWM and 190 matches to the M11 PWM in the entire genome; at a lower stringency (1.00/0.95), there are 1198 matches to the M1 PWM and 703 matches to the M11 PWM (**Supplementary Table S6**). Thus, the ∼200 copies of these motifs found in the 337 ZNF274 peaks represents a significant fraction of the motifs encoded in the human genome. Finally, we performed a location analysis of the M1 and M11 matches in the human genome and found that 80% of the matches were at ZNF genes; for M1: 569 of 724 top matches and 968 of 1198 lower stringency matches; for M11: 148 of 190 top matches and 571 of 703 lower stringency matches. Thus, the M1 and M11 motifs are essentially only found in or near ZNF genes.

Although the entire set of ZNF274 binding sites were used for the motif analysis, the two long motifs shown in **Figure S8, panel A** are highly enriched in the top 100 ranked peaks. In fact, both motifs are present in 72 of the top100 ranked ZNF274 binding sites. This motif enrichment is similar to the enrichment of motifs under ChIP-seq peaks for other well-characterized factors [42]. The fact that both motifs are often found in the same binding site lead us to examine the relationship of the two motifs. We found that the 3′ end of M11 is identical to the 5′ end of the reverse orientation of M1 (**Figure S8**, **panel B**). These two motifs due in fact constitute a single long motif in a large number of the ZNF274 binding sites (see **Figure S8**, panel C and **Figure S10**). Interestingly, the 29 mer encodes the linker and conserved H2 helix that are found in C2H2 ZNFs (**Figure S8, panel D**). It is important to note that not all C2H2 zinc fingers contain the 29mer (we used C2H2 zinc fingers that are not bound by ZNF274 as our background model to identify the M1 and M11 motifs; see also **Figure S10**). Sequence analysis indicates that the C2H2 ZNFs that do not contain the 29mer use different nucleotides to encode their linker and H2 helix.

### ZNF274 recruits the KAP1/SETDB1 epigenetic regulatory complex to the human genome

**Figure 7:**
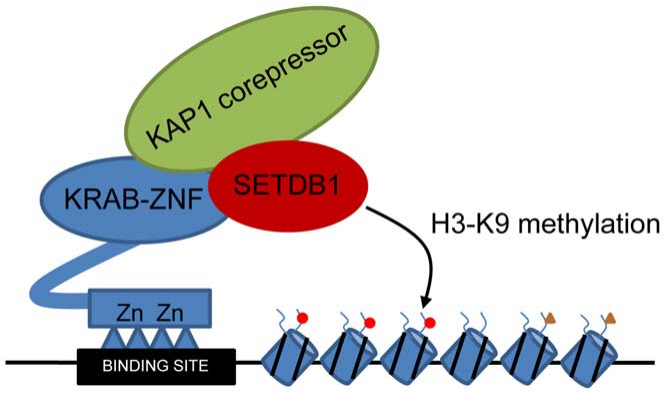
A model for SETDB1 recruitment. Shown is a schematic illustrating the current model for KAP1-mediated transcriptional repression. KAP1 is thought to be brought to the genome by interaction with a KRAB-ZNF, which binds site-specifically to DNA. KAP1 in turn is thought to recruit the histone methyltransferase SETDB1, which then specifically mediates trimethylation of lysine 9 of histone H3 near the KRAB-ZNF binding sites.

**Figure 8:**
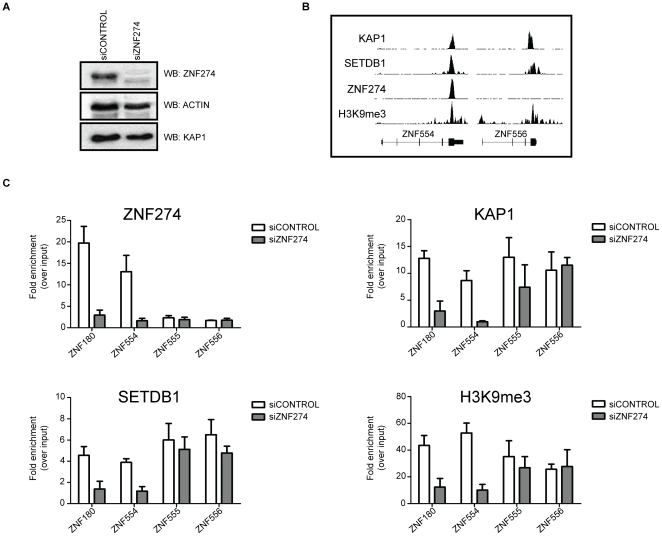
Depletion of ZNF274 inhibits KAP1 recruitment and H3K9 trimethylation at target genes. A) Control siRNAs or specific siRNAs targeting ZNF274 were transfected into HelaS3 cells and western blot analysis of ZNF274 was performed; KAP1 and Actin antibodies were used as loading control. B) Shown are the binding patterns of KAP1, SETDB1, ZNF274, and H3K9me3 on a ZNF274 target gene (*ZNF554*) and on a zinc finger gene not bound by ZNF274 (*ZNF556*). C) Control siRNAs or specific siRNAs targeting ZNF274 were transfected into HelaS3 cells and ZNF274, KAP1, SETDB1 and H3K9me3 chromatin immunoprecipitation (ChIP) analyses were performed. The fold change in occupancy of ZNF274, KAP1, SETDB1, and H3K9me3 at the ZNF274 positive targets (*ZNF180* and *ZNF554*) and negative sites (*ZNF555* and *ZNF556*) for each factor was measured as the change in the mean enrichment for that factor from experiments with siRNA against ZNF274 over control siRNAs.

Based on the similar binding patterns of ZNF274, KAP1, and SETDB1 and a current model in the field that a KRAB-ZNF recruits SETDB1 and KAP1 to the genome (**Figure 7**), we hypothesized that ZNF274 is a site-specific factor that recruits the SETDB1 histone methyltransferase to specific regions of the genome. To test this hypothesis, we knocked down the levels of ZNF274 using transfection of siRNAs. As shown in **Figure 8A**, protein levels of ZNF274 were reduced when siRNAs specific to ZNF274 mRNA were introduced into cells. To determine the effects of loss of ZNF274, we performed ChIP analysis on two ZNF274 binding sites (*ZNF180* and *ZNF554*) and on two ZNFs that are not ZNF274 binding sites (*ZNF555* and *ZNF556*); see **Figure 8B** for binding profiles of KAP1, SETDB1, ZNF274, and H3K9me3 for *ZNF554* (a ZNF274 binding site) vs. *ZNF556* (not a ZNF274 binding site). We found that introduction of siRNAs to ZNF274 reduced the ZNF274 ChIP signal at the two binding sites but did not affect the extremely low signals at the non targets *ZNF555* and *ZNF556* (**Figure 8C**). The KAP1, SETDB1 and H3K9me3 signals were also reduced at *ZNF180* and *ZNF554*, but not at *ZNF555* or *ZNF556*. These results are consistent with the hypothesis that, at specific sites, ZNF274 recruits KAP1, which recruits SETDB1, which results in trimethylation of histone H3 on lysine 9.

## Discussion

### The H3K9me3 methylome

**Figure 9:**
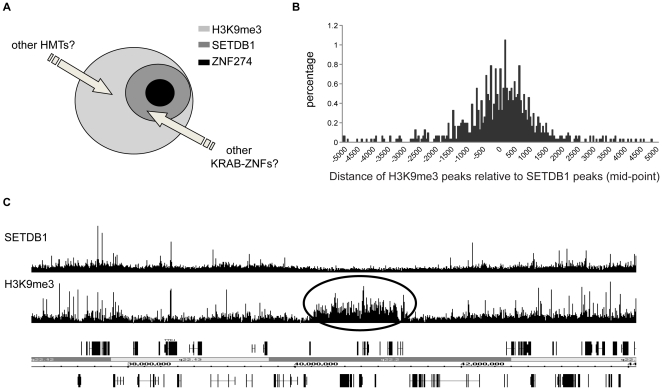
The H3K9me3 methylome. A) Schematic showing that the histone methytransferase SETDB1 is responsible for a large proportion, but not all, of the H3K9me3 sites in K562 cells and that ZNF274 is responsible for recruiting SETDB1 to some, but not all, of the SETDB1 binding sites. B) The distance of all H3K9me3 peaks found in the top 10% of that dataset is shown relative to the center of the nearest SETDB1 peak. C) Shown is the binding pattern of SETDB1 and H3K9me3 for a region of chromosome 21, indicating that there are large regions of the genome covered by H3K9me3 but not by SETDB1.

We have previously shown that homeobox genes are bound by H3K27me3 whereas the 3′ regions of zinc finger genes are bound by H3K9me3 [2], [3], [10]. The overall goal of this current study was to determine the mechanism by which a H3K9me3-specific histone methyltransferase is recruited to the 3′ ends of C2H2 zinc finger genes. To achieve this goal, we first demonstrated that SETDB1 colocalizes with H3K9me3 at zinc finger genes, then we identified ZNF274 as a DNA binding factor that can recruit the KAP1/SETDB1 histone methyltransferase complex to the 3′ ends of certain zinc finger genes. Thus, ZNF274 is a critical component of this epigenetic complex in a variety of cell types. However, KAP1 and SETDB1 are recruited to additional zinc finger genes than are not bound by ZNF274 (see **Figure 8B**), indicating that at least one other factor (likely a KRAB-ZNF protein) must be involved with recruitment of KAP1/SETDB1 to H3K9me3 sites. We have also shown that H3K9me3 is found at a large number of sites that are devoid of KAP1 and SETDB1. However, one must keep in mind the fact that H3K9me3 displays a spreading, not a peak-like, ChIP-seq pattern at many sites. It is possible that a single recruitment of SETDB1 could result in the methyation of several nucleosomes on either side of a binding site. To test this hypothesis, we plotted the position of the top 10% (3047 peaks) of the H3K9me3 sites relative to the center of the peak of the 3238 SETDB1 peaks. As shown in **Figure 9B** **,** most of the strongest H3K9me3 sites are located within 1-2 kb of a SETDB1 site, indicating that SETDB1 is an important contributor to the H3K9me3 regulome. However, we note that there are regions of the genome that show large coverage by H3K9me3 but no SETDB1 binding (**Figure 9C**). Thus, the H3K9me3 regulome must be defined by both SETDB1 and another, as yet unidentified, histone methyltransferase (**Figure 9A**). Other candidate histone methyltransferases include G9a/KMT1C, GLP/KMT1D, Suv39h1/KMT1A and Suv39h2/KMT1B [21], [22], [23], [24], [25], [26]. Our analysis of the binding patterns of G9a suggest that it is not involved in H3K9me3 formation on chromosome 19. Our findings are supported by previous studies linking G9a to mono and dimethylation (but not trimethylation) of lysine 9 of histone H3 [45], [46]. However, we also note that other studies have shown that reduction of G9a can have modest effects on global H3K9me3 levels [47]. Also, a recent study has shown that a subset of G9a/KMT1C, GLP/KMT1D, Suv39h1/KMT1A and SETDB1/KMT1E exist in a large complex and that reduction of G9a can result in a reduction of H3K9me3 at certain locations [48]. Perhaps the regions (e.g. centromeres or repetitive elements) at which G9a modulates H3K9me3 are not easily studied by ChIP-chip and/or they are not located on chromosome 19. We have not been successful in enriching for H3K9me3-bound regions using a variety of commercially available antibodies to Suv39h1/KMT1A or Suv39h2/KMT1B (data not shown), but it is not possible to draw conclusions from negative results because the antibodies may not have been of high enough affinity for these experiments.

As one way of addressing whether there are distinct subsets of H3K9me3-bound regions, we performed gene ontology analyses. We divided the H3K9me3 sites into sets bound by H3K9me3 and SETDB1 vs. sets bound by H3K9me3 but not by SETDB1. Using the Database for Annotation, Visualization and Integrated Discovery (DAVID) [49], [50], we first performed a gene ontology analysis of the sites bound by both H3K9me3 and SETDB1. As expected, based on our previous studies [2], [3], [10], we found a striking enrichment of gene categories related to transcription and DNA binding. In contrast, when we analyzed the sites bound by H3K9me3 but not by SETDB1, we observed enrichment in numerous different functional categories including genes involved in signaling through phosphorylation, amino acid transport, cell adhesion, metabolism and development (using a p-value <1e-3; see **Supplementary Table S6**). These annotations imply broad functions for H3K9me3 and are consistent with reports that H3K9me3 is present across a wide range of genes [51], [52] and that it may be involved in global organization rather than a direct role in gene regulation [53]. Our studies highlight the concept that specific subsets of H3K9 trimethylated regions exist across the genome and support a specific role for SETDB1 in altering the chromatin structure of zinc finger transcription factor genes through H3K9 methylation.

We also note that a recent paper has implicated SETDB1 in maintenance of the mouse ES cell pluripotent state [54]. In that study, SETDB1 was found mainly at promoters of genes involved in developmental regulation and many of those promoter regions were also occupied by H3K9me3, H3K27me3, and SUZ12. These results are in direct contrast with our studies showing that SETDB1 binding sites are the 3′ ends of ZNF genes (not promoters) and that they correlate with H3K9me3 but not H3K27me3. Additionally, we find no binding of SUZ12 to SETDB1 binding sites in K562 cells (data not shown). These results suggest that SETDB1 plays a unique role in the more differentiated human K562 cells as compared to its role in pluripotent mouse ES cells.

### Specificity of binding of ZNF274

As indicated above, ZNF274 is specifically targeted to 3′ ends of a subset of ZNF genes. In fact, the few binding sites that have a “nearest” gene that is not a ZNF, such as the site located in intron 4 of the MAPK9 gene, are actually evolutionary remnants of ZNF genes (as determined by DNA sequence similarity identified using BLAT). However, not all ZNF 3′ ends are bound by ZNF274. Although one could certainly imagine chromosomal positioning influencing accessibility of ZNF274 to binding sites, there are many examples where ZNF274 binds to the 3′ end of one ZNF in a cluster but not to the neighboring ZNFs. For example, the nearest neighbors to the ZNF554 3′ end (which is bound by ZNF274) are ZNF555 and ZNF556. However, the ZNF555 and ZNF556 3′ exons are bound by KAP1 and SETDB1 but not by ZNF274. Thus, there must be some degree of specificity to the binding of ZNF274. Using the prediction method described in Pabo et al. [55], the zinc finger code predicts a fairly non-specific binding site for ZNF274 of 5′NGAGGAANCANANNN3′ or the complement 5′NNNTNTGNTTCCTCN3′ (DJ Segal, unpublished information). However, these sites are not similar to the motifs identified in the ChIP-seq identified regions. It remains to be determined exactly how specificity of binding is achieved by ZNF274 but possibilities include heterodimerization with another protein or selective use of a subset of the zinc fingers.

### Conclusion

Our studies have provided the first support for the working model that an endogenous KRAB-ZNF (ZNF274) can, within a cellular environment, recruit the KAP1/SETDB1 histone methyltransferase complex to the genome and that these sites correspond to sites of H3K9me3. However, because ZNF274 does not bind to all ZNF 3′ ends, our studies also clearly suggest that additional KRAB-ZNFs contribute to the formation of the H3K9me3 methylome. Future ChIP-seq studies of additional candidate KRAB-ZNFs as well as mass spectrometry to identify additional SETDB1 partners are required to identify the set of KRAB-ZNFs involved in H3K9me3 pattern formation. Finally, we are continuing to investigate H3K9me3 sites that are not bound by KAP1/SETDB1 with the goal of identifying other involved histone methyltransferases.

## Materials and Methods

### Cell culture

Human chronic myelogenous leukemia cells (K562, ATCC # CCL-243) and human lymphoblastoid cells (GM12878, Coriell Catalog ID GM12878) were grown in RPMI supplemented with 10% FBS, 2 mM L-Glutamine, 100 U/mL Pen-Strep. Human cervical carcinoma cells (HeLa-S3, ATCC CCL-2.2) and human hepatocellular carcinoma (HepG2, ATCC # HB-8065) were grown in Dulbecco's modified Eagle's medium supplemented with 10% FBS, 2 mM L-Glutamine, 100 U/mL Pen-Strep.

### ChIP-chip assays

TRIM28 (KAP1), G9a, H3K9me3, and SETDB1 ChIP samples using 10^7^ K562 cells per ChIP were prepared as described at http://www.genomecenter.ucdavis.edu/farnham/protocol.html with minor modifications. ChIP assays were performed using 1.6 µg SETDB1 antibody (Proteintech Group #11231-1-AP), 5 µg G9a antibody (Upstate #07-551) or 2 µg TRIM28 (KAP1) antibody (Abcam #ab10483). Two G9a ChIPs and three KAP1 ChIPs were pooled and concentrated using speedvac before amplification. Prior to array hybridization, TRIM28 (KAP1), SETDB1 and G9a ChIP DNA was amplified using the Sigma GenomePlex WGA2 kit as described in O'Geen et al. [56]. Briefly, the initial random fragmentation step is omitted and DNA from the entire ChIP sample(s) or from 20 ng input DNA is amplified. TRIM28 (KAP1) and SETDB1 amplicons were confirmed by PCR using primers for ZNF333 as a positive control and for hPolII as a negative control. Labelling of amplicons and hybridization to chromosome tiling arrays were performed by Roche NimbleGen. The HG18Tiling Set 9 HX1 of the NimbleGen HD2 platform was used, which contains chromosomes 17, 18, and 19 and parts of chromosomes 16 and 20 tiled at a 100 bp interval using probes with lengths between 50-75 bp with a target Tm of 76°C. All data is MIAME compliant and the raw data has been deposited at the MIAME compliant Gene Expression Omnibus (GEO) database at National Center for Biotechnology Information (http://www.ncbi.nlm.nih.gov/geo) and are accessible through accession number GSE24480.

**ChIP-chip peak calling:** Oftentimes oligo intensities will vary within a binding site due to lack of sensitivity in hybridization. As a result, many ChIP-Chip peak-calling programs currently available will not detect certain peaks, and perform poorly with histone modification data. Therefore, in order to interpret our data more accurately, we have developed a new ChIP-chip peak-calling program, Mayacamas (available http://www.genomecenter.ucdavis.edu/farnham/software.html). The data was first smoothed using a sliding average: the values of five oligos on either side of a center oligo are averaged to determine the new smoothed value of the center oligo. Using the smoothed data, the top 5%, top 1% and top .5% of oligos were determined. If adjacent oligos, spanning at least 800 bp, were above threshold, the region was called a peak. We accounted for the fact that ChIP-Chip arrays often lack oligos in certain regions that are not unique by allowing for 6 or less missing oligos in a peak region. In order to eliminate false positives produced by this, we also specified that a peak region must include at least half of the number of expected oligos. We used the peak cutoff of top 5% for H3me3K9 in order to account for the spreading nature of histone marks. The top 1% cutoff was used for KAP1 and SetDB1 experiments, and the top .5% cutoff was used for G9a to eliminate false positives since this factor does not have many binding sites.

### CHIP-seq assays

For ChIP-seq, KAP1 and ZNF274 ChIP samples were prepared from K562 cells as follows: cultures of 1×10^8^ K562 cells were harvested at a density of 10^6^ cells/mL and cross-linked with 1% formaldehyde for 10 minutes at room temperature. Cross-linking was stopped by the addition of glycine to 125 mM final concentration and cells were washed twice with 1×PBS. The cell pellet was then resuspended in 2 mL ChIP lysis buffer (50 mM Tris-Cl pH 8.0, 5 mM EDTA, 1% SDS, 1 complete protease inhibitor tablet [Roche]) and incubated on ice for 30 minutes. Samples were sonicated for 30 minutes, with 30-second pulses, 1 minute resting, using the Bioruptor sonicator (Diagenode) to produce chromatin fragments of 0.5 kb on average. After clarification by centrifugation, sonicated extracts were precleared with 20 µl/10^7^ StaphA cells blocked with 10 mg/mL BSA. The precleared extracts were diluted 1∶10 with ChIP dilution buffer (1% triton X-100, 2 mM EDTA, 150 mM NaCl, 20 mM Tris-Cl pH 8.0, 5 mM PMSF). Anti-ZNF274 antibody or anti-KAP1 antibody (Novus Biologicals, catalog # H00010782-A01 and AbCam, catalog # ab3831, respectively) was added at a concentration of 2 µg/10^7^ cells and incubated for 12 hours at 4°C. A rabbit anti-mouse or rabbit anti-goat secondary antibody was added at an equal concentration for 1 hour at 4°C. Complexes were recovered with StaphA cells for 15 minutes at room temperature and washed 5 times with ice cold RIPA buffer. Precipitates were resuspended in 100 µl ChIP elution buffer (1% SDS, 0.1 M NaHCO_3_), incubated at 65°C for 12 hours and treated with 10 µg RNaseA for 20 minutes at 37°C. After pooling, the DNA was recovered from the eluate using the QIAquick PCR Purification kit (QIAGEN) according to the manufacturer's instructions. For H3K9me3 ChIP-seq, chromatin was prepared as above, except only 5×10^6^ K562 cells were used and 5 µg anti-H3K9me3 antibody (Diagenode, catalog # pAb-056-050) was added to the ChIP reactions. For SETDB1 ChIP-seq, ChIP samples were prepared as for ZNF274 and KAP1 from 10^8^ K562 cells, using 20 µg anti-SETDB1 antibodies (Proteintech Group, catalog # 11231-1-AP), except chromatin was prepared using the SimpleChIP™ Enzymatic Chromatin IP Kit according to the manufacturer's recommendations (Cell Signaling Technology, catalog # 9003). For ZNF274 ChIP-seq assays using HeLa, GM12878 and HepG2 cells, the same conditions for K562 cells were used. For ChIP assays using siRNA-transfected cells, HeLa cells were transfected with siRNAs (see below) and chromatin was prepared by sonication. Antibodies specific for ZNF274, KAP1, SETDB1 and H3K9me3 were used at 1 ug/2.5×10^5^ cells. All ChIP-seq data is deposited in the Gene Expression Omnibus (GEO) database at National Center for Biotechnology Information (http://www.ncbi.nlm.nih.gov/geo) and are accessible through accession number GSE24632.

### ChIP-seq library construction and quantitation

ChIP libraries were created as described previously [57], using 15 cycles of amplification. Libraries were run on a 2% agarose gel and the 200-400 and 400-600 fractions of the library was extracted and purified. To estimate the yield of library and its relative amplification value, library DNA was quantitated using a Nanodrop and serial dilutions of 1.25 nM library were compared to a reference library by real-time PCR using primers complementary to the library adapters. In general, we have found that the 400-600 size libraries contain higher enrichments for heterochromatic regions and for factors that bind to H3K9me3 (see **Supplementary Figure S3**). The amplification value relative to the reference library was used to estimate the flowcell loading concentration. The ChIP-seq libraries were run on an Illumina GA2 by the DNA Technologies Core Faciity at the University of California-Davis (http://genomecenter.ucdavis.edu/dna_technologies/).

### ChIP-seq peakcalling

The Sole-search software was modified in order to more accurately identify histone regions. Duplication and deletion events were first determined in the input datasets and the sequenced ChIP and background files were normalized accordingly, as described in the original version [29]. In the new modified version of Sole-search (described in Blahnik et al. in preparation), both the ChIP-seq data and the background model are smoothed, before determining a statistically significant peak height cutoff. Data was binned using a sliding window of 30 bp. The value for each bin was calculated as the mean of all values within 600 bp upstream and 600 bp downstream of the bin. Statistically significant height cutoff was determined as the lowest value not surpassing a false discovery rate of 0.01. The final of the three steps in Sole-search, significance over background, was removed because small peaks in input data do not affect peak-calling when analyzing ChIP-seq data composed of broad chromatin domains, rendering this step unnecessary. Peaks were called if they exceeded height cutoff and length cutoff. Minimum length was determined as 150 bp, approximating the minimal chromatin fragment size used in a library procedure.

### Sequential ChIP assays

Crosslinked cell extracts were prepared from 2×10^7^ HeLa cells as described above. The extracts were split into six separate ChIP reactions, three for ZNF274 ChIPs and three for KAP1 ChIPs using 2 µg antibody per reaction. The primary ChIP reactions were incubated for 12 hours at 4°C, washed and eluted as described above. The eluates were then diluted 1∶10 with ChIP dilution buffer for sequential ChIP reactions using 1 µg of the indicated antibody. The sequential immunoprecipitations were incubated for 6 hours at 4°C, washed five times with ice cold RIPA buffer and eluted in 100 µL ChIP elution buffer. The eluted DNA was purified using Qiagen PCR purification columns and used for PCR reactions with 37 cycles of PCR.

### Quantitative real-time PCR

Quantitative real-time PCR (qPCR) was performed on a Bio-Rad DNA Engine Opticon Real-Time PCR System using SYBR® Green Master PCR Mix according to the manufacturer's instructions (Invitrogen). The fold enrichment of each target site was calculated as 2 to the power of the cycle threshold (cT) difference between input chromatin and ChIP samples. All primers are listed in **Supplementary Table S5**.

### siRNA treatment

For ZNF274 knockdown RNA analysis, HeLa cells were transfected with 40 nM ZNF274 siRNAs (Stealth Select RNAi; Invitrogen, HSS116638;HSS116639;HSS116640) or si-GLO RISC-Free (Dharmacon, cat# D-001600-01) as a non-specific control using Invitrogen Lipofectamine2000 according to manufacture recommendations. Cells were transfected, retransfected 48 h later and harvested at 96 h following the initial transfection for collection of RNA, protein or chromatin. RNA was prepared using Invitrogen Trizol according to the manufacturer's recommendations; protein was extracted with ice-cold RIPA buffer containing a protease inhibitor cocktail and chromatin was prepared as described.

### GST-KRAB domain purification

cDNA fragments encoding the KRAB domains of human ZNF10 (residues 2-90), ZNF263 (residues 206-299) and ZNF274 (residues2-90 for KRAB1 and residues 278-366 for KRAB2) were amplified by PCR and subcloned into the pGEX-SG vector. The purification of recombinant GST-KRAB proteins was performed using glutathione agarose beads according to the manufacturer's recommendations (Invitrogen). Briefly, *Escherichia coli* BL21 Star cells (Invitrogen, catalog # C6010-03) bearing the plasmid were propagated with aeration at 37°C in 250 mL of Luria broth to an *A*_600_ of approximately 0.6. IPTG was added to 1 mM, and growth at 37°C was continued for four hours. The cells were harvested by centrifugation washed once with ice-cold PBS and resuspended in phosphate buffered saline (PBS) supplemented with 0.1% Triton X-100, 1 mM PMSF, 5 mM DTT, 100 mM MgCl_2_ and 0.5 mg/ml lysozyme. The cell suspension was incubated for 1 hour at 4°C with slow mixing. The suspension was then snap frozen and quick thawed to lyse the cells. Following brief sonication, cell debris was removed by centrifugation at 10 000×g at 4°C for 30 min. The supernatant was mixed with 0.2 mL glutathione agarose beads (washed twice in PBS) and incubated for 1 hour at 4°C with gentle mixing. The beads were pelleted and washed twice times with PBS containing 0.1% Triton X-100 and 2 M NaCl, twice with PBS containing 0.1% Triton X-100 and once in 10 mM Hepes-NaOH (pH 7.9), 0.3 M KCl, 1.5 mM MgCl2, 0.5 mM PMSF and 0.1% Triton X-100.

### KAP1 interaction assay

Recombinant GST-KRAB domains or GST alone prebound to glutathione-agarose beads in 10 mM Hepes-NaOH (pH 7.9), 0.3 MKCl, 1.5 mM MgCl2, 0.5 mM PMSF, and 0.1% Triton X-100 was incubated with undialyzed nuclear extracts for 4 hours at 4°C prepared from 1×10^7^ K562 cells according to the method of Dignam [58]. The glutathione-agarose beads were then washed 7 times with the same buffer containing 0.5 M KCl, and bound proteins were eluted from the beads with 100 mM Tris-HCl (pH 7.9), 0.12 M NaCl, 0.1%Triton X-100, and 20 mM glutathione. The eluates were analyzed by 10% SDS-PAGE, and proteins were visualized by coomassie staining and western blotting with antibodies specific to KAP1.

### Co-immunoprecipitation assays

A total of 2×10^8^ K562 cells were lysed in buffer A containing 20 mM Tris-HCl (pH 8.0), 150 mM NaCl, 2.5 mM EDTA, 0.5% NP-40, 5% glycerol, 0.2 mM phenylmethylsulfonyl fluoride (PMSF), and 0.5 mM dithiothreitol. Cell lysates were precleared with protein G-agarose beads for 1 hour at 4°C and then incubated with the indicated antibody for 5 hours at 4°C with rotation at 5 µg of antibody per immunoprecipitation. The reactions were precipitated with 40 µL protein G-agarose beads for 1 hour at 4°C and then washed five times with lysis buffer and eluted by boiling in sample buffer. The eluted proteins were resolved on 10% SDS-PAGE gel electrophoresis for western blotting analysis using the indicated antibodies.

### RNA data analysis

Affymetrix Human Exon 1.0 ST (HuEx-1_0-st-v2) Arrays, consisting of 1.4 million probe sets of clustering 1.0 million exon clusters from >5,500,000 features, were used for measuring basal level gene expression in K562 cells. A total of seven biological samples from seven different institutions (from seven ENCODE consortium groups) were performed on this array platform and the generated quantified Affymetrix image files (seven *.CEL files) were analyzed simultaneously and normalized using the RMA algorithm provided in the Expression Console software developed by Affymetrix Inc. Probe sets were annotated as supported by RefSeq and full-length GenBank Transcripts, resulting in 21,924 annotated probe sets representing ∼18,000 annotated genes. A scale of LOG2 was used for calculating the abundance for each probe set gene for each sample. The RNA expression array data can be accessed through the NCBI Gene Expression Omnibus database at National Center for Biotechnology Information through the accession number GSE19146.

We computed a mean value of seven replicates for each gene on the array as a final value of the gene's expression level. We then compared the expression levels of the ZNF274 target genes, as identified using a location analysis program. We note because most location analysis programs link a binding site with the nearest transcription start site, we had to modify our location analysis program to allow appropriate characterization of ZNF274 binding sites. ZNF274 binds to the 3′ exons of C2H2 zinc finger genes and, due to the high density of genes on chromosome 19, the binding site is in many cases closer to the start site of the neighboring gene than it is to the start site of the gene to which the exon belongs. Standard location analysis programs would incorrectly identify ZNF274 binding sites as being in proximal promoter regions, when in fact they are located in 3′ exons. Therefore, to identify the “nearest” gene to the ZNF274 binding sites, our location analysis program was modified to first identify binding sites within genes and then to look for the nearest gene for those sites not found in the first pass. After obtaining the list of genes nearest to ZNF274 binding sites, we determined their expression level in K562 cells.

### De novo motif analysis

To identify motifs enriched under ZNF274 binding sites from the K562 ChIP-seq data, we first retrieved 100 nt from each of the 337 binding sites (50 nt on either side of the mid-point of the peak). Then, we applied the de novo motif discovery approach (ChIPMotifs) which can identify both known and novel motifs [43], [44]. Although the standard background data set for motif finding is promoter regions, this was not appropriate for the ZNF274 peak analysis. The ZFN274 peaks are all located within the 3′ coding exons of ZNF genes and, as such, all contain common motifs such as finger domains and linker domains that may be related to the coding potential of the exon and not to the recruitment of ZNF274. Therefore, we used a set of ∼90 sequences that represented 3′ exons from ZNF genes that were not bound by ZNF274 as the negative dataset. Using this apprpoach, we identified a total of 28 motifs, none of which have a significant match to any known motif in the TRANSFAC [59] and JASPAR [60] databases. From the 28 identified motifs, clusters of similar motifs were derived based on sequence similarity (many of the shorter motifs were contained within longer motifs).

## Supporting Information

Figure S1 Identification of G9a target sites in K562 cells using tiling arrays.
A. ChIP-chip analysis of G9a binding sites.
B. PCR analysis of G9a binding sites. Using separate biological ChIP replicates, three G9a binding sites identified by ChIP-chip were confirmed to be G9a, but not SETDB1, targets. In contrast, the H3K9me3 target ZNF84 shows the presence of SETDB1, but not G9a. As expected, the active MFAP promoter is not occupied by either of the histone methyltransferases. Enrichments are shown in comparison to total chromatin DNA; IgG was used as a negative control.
(PDF)

Figure S2 Western blot and immunoprecipitation-western blot validation of anti-ZNF274 antibody. A) Western blot using 30 ugnuclear extracts prepared from 4 human cell lines. B) Immunoprecipitationof ZNF274 from HepG2 nuclear extracts using control rabbit IgG and anti-ZNF274 mouse IgG. The signal from the mouse anti-ZNF274 IgGis indicated.
(PDF)

Figure S3 qPCR analysis of ZNF274 ChIP-seq libraries prepared from two different fractions. ZNF274 libraries were prepared as described in the Materials and Methods section. Following 14 cycles of PCR amplification and agarose gel electrophoresis, two different sized fractions (200-400 bp and 400-600 bp) were excised and extracted and compared to a library of input non-ChIP enriched DNA. The targets analyzed are shown below and primer sequences are listed in the supplementary information.
(PDF)

Figure S4 Enriched motifs found in ZNF274 binding sites. W-ChIPMotifs was used to extract enriched motifs present in ZNF274 binding sites as described in the Materials and Methods section. Shown are the detected motifs with their SeqLOGOs, PWMs, core and PWM scores, *P*-values and Bonferroni correction *P*-value at different percentile levels for each identified motif.
(PDF)

Figure S5 The similarity of the ZNF274 enriched motifs is compared to the closest known motif for other factors using the STAMP tool. Shown are the matched similar motifs from the STAMP tool.
(PDF)

Figure S6 Comparison of peak sets identified using ChIP-chip and ChIP-seq for H3K9me3, SETDB1, and KAP1. An example of the binding patterns for H3K9me3, SETDB1, and KAP1 using the different platforms is shown in panel A. The ChIP-chip vs. ChIP-seq binding patterns for H3K9me3, for SETDB1, and KAP1 are very similar. However, the ChIP-chip data has considerably more background and the data is compressed into a smaller range (such that it is difficult to distinguish the very highest peaks from the moderately high peaks). For H3K9me3, we identified 1497 ChIP-chip peaks and 3866 ChIP-seq peaks on chromosome 19, and found that half of the H3K9me3 peaks from obtained using one platform are represented in the dataset obtained using the other platform (panel B); in general, these common peaks are found in regions displaying the highest and widest coverage by H3K9me3. The peaks that did not overlap were narrower peaks that tended to be difficult to analyze in one of the experimental platforms. For example, some regions were not represented on the array because of the spacing of the oligonucleotides and some regions were not represented in the sequenced tags because they matched to more than one place on the genome. We next compared the SETDB1 and KAP1 peak sets obtained using ChIP-chip vs. ChIP-seq. Again, the ChIP-seq data was much more robust, having higher signal to noise than the ChIP-chip data. Most of the SETB1 (83%) and KAP1 (95%) ChIP-seq peaks were contained within the ChIP-chip peak sets, suggesting that the extra peaks in the ChIP-chip sets are false positives.
(PDF)

Figure S7 Heatmap of expression data for ZNF274 targets. The expression levels of the subset of ZNF274 target genes (identified as the nearest gene to each binding site from the K562 ZNF274 ChIP-seq dataset) present on the Affymetrix Human Exon 1.0 ST (HuEx-1_0-st-v2) Arrays is shown compared to the expression levels of the same number of genes from each of the 5 quintiles representing expression of all mRNAs in K562 cells.
(PDF)

Figure S8 Motifs identified inZNF274 binding sites. (**A**) Motifs identified within 50 nucleotides of either side of the center of ZNF274 binding sites. Also indicated is the number of occurrences of each motif in the 337 ZNF274 binding sites identified in K562 cells. Both ZNF274M11 and ZNF274M1 are present in 72 of the top 100 ranked ZNF274 binding sites from K562 cells. (**B**) The overlap of the 5′ end of the reverse orientation of the M1 motif and the 3′ end of the M11 motif. (**C**) The sequence of the full 29mer. (**D**) The reverse orientation of the 29mer encodes the conserved linker and the conserved H2 helix found in C2H2 zinc fingers.
(PDF)

Figure S9 ChIP-seq binding patterns of ZNF274, KAP1, SETDB1, and H3K9me3 at individual gene loci. Shown are snapshots of the ChIP-seq data at specific C2H2 ZNF genes.
(PDF)

Figure S10 Shown is a comparison of ZNF274 binding sites, the location of all C2H2 ZNF genes, and the location of the identified 29mer motif (with 2 allowed mismatches) in forward and reverse orientations across the right arm of chromosome 19. There is a high correspondence between ZNF274 binding sites and the subset of C2H2 ZNF genes that contain the 29mer motif.
(PDF)

Table S1 A summary of the ChIP-chip and ChIP-seq datasets from this study.
(XLS)

Table S2 ChIP-chip peak sets for SETDB1, KAP1, H3K9me3, and G9a.
(XLS)

Table S3 ChIP-seq peak sets for ZNF274 in the cell lines K562, HeLa, HepG2, and GM12878.
(XLS)

Table S4 ZNF274 motif distribution.
(XLS)

Table S5 Primers used for this study.
(XLS)

Table S6 A list of the target genes for the top 10% of the H3K9me3 peaks either containing SETDB1 peaks or not having a SETDB1 peak. Also shown is the GO ontology analysis for biological process for those target genes.
(XLS)
