# Supplementary material for: ZNF274 Recruits the Histone Methyltransferase SETDB1 to the 3′ Ends of ZNF Genes
Source: PLoS One. 2010 Dec 8;5(12):e15082. doi: 10.1371/journal.pone.0015082 (PMC2999557; doi:10.1371/journal.pone.0015082)
Supplement: Figure S1 — Identification of G9a target sites in K562 cells using tiling arrays. A. ChIP-chip analysis of G9a binding sites. B. PCR analysis of G9a binding sites. Using separate biological ChIP replicates, three G9a binding sites identified by ChIP-chip were confirmed to be G9a, but not SETDB1, targets. In contrast, the H3K9me3 target ZNF84 shows the presence of SETDB1, but not G9a. As expected, the active MFAP promoter is not occupied by either of the histone methyltransferases. Enrichments are shown in comparison to total chromatin DNA; IgG was used as a negative control. (PDF) [file pone.0015082.s001.pdf]

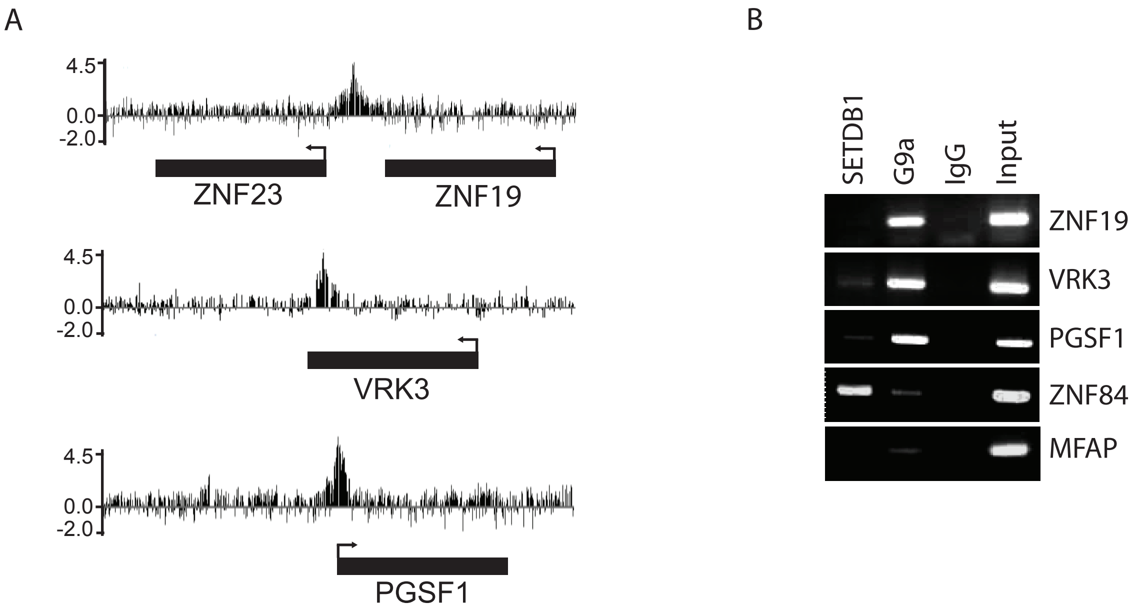

Frietze_Figure S1. Identification of G9a target sites in K562 cells using tiling arrays.
A. ChIP-chip analysis of G9a binding sites.
B. PCR analysis of G9a binding sites. Using separate biological ChIP replicates, three G9a binding sites identified by ChIP-chip were confirmed to be G9a, but not SETDB1, targets. In contrast, the H3K9me3 target ZNF84 shows the presence of SETDB1, but not G9a. As expected, the active MFAP promoter is not occupied by either of the histone methyltransferases. Enrichments are shown in comparison to total chromatin DNA; IgG was used as a negative control.
