# Supplementary material for: ZNF274 Recruits the Histone Methyltransferase SETDB1 to the 3′ Ends of ZNF Genes
Source: PLoS One. 2010 Dec 8;5(12):e15082. doi: 10.1371/journal.pone.0015082 (PMC2999557; doi:10.1371/journal.pone.0015082)
Supplement: Figure S2 — Western blot and immunoprecipitation-western blot validation of anti-ZNF274 antibody. A) Western blot using 30 ugnuclear extracts prepared from 4 human cell lines. B) Immunoprecipitationof ZNF274 from HepG2 nuclear extracts using control rabbit IgG and anti-ZNF274 mouse IgG. The signal from the mouse anti-ZNF274 IgGis indicated. (PDF) [file pone.0015082.s002.pdf]

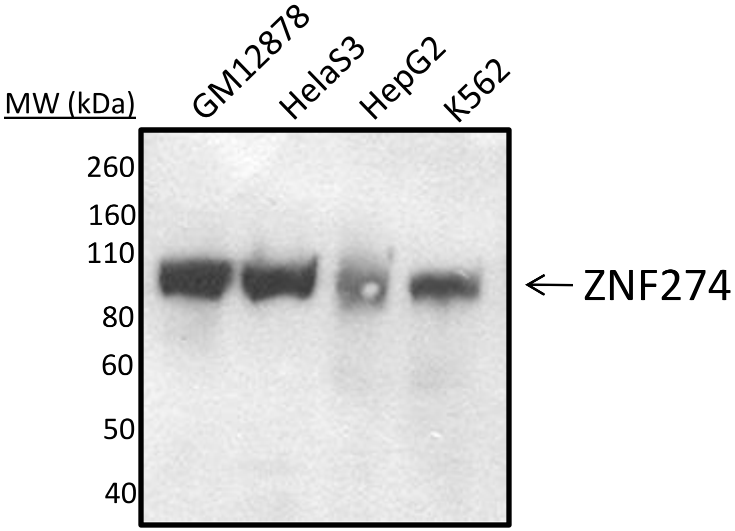

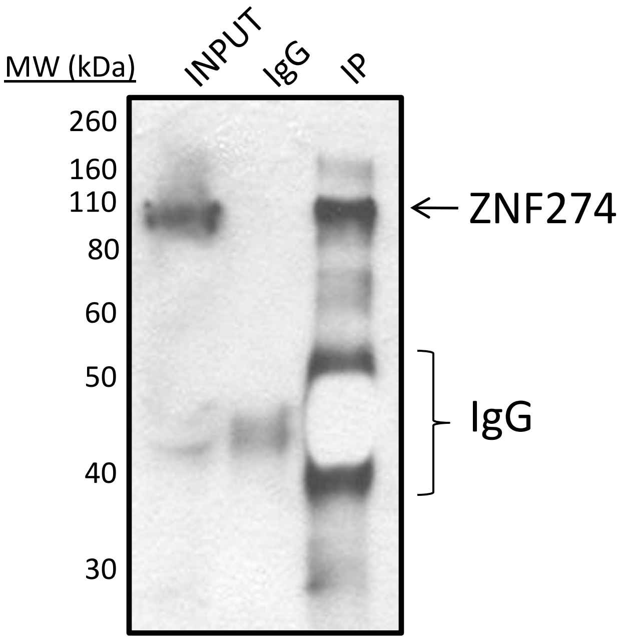

Frietze_Figure S2. Western blot and immunoprecipitation-western blot validation of anti-ZNF274 antibody. A) Western blot using 30 ug nuclear extracts prepared from 4 human cell lines. B) Immunoprecipitation of ZNF274 from HepG2 nuclear extracts using control rabbit IgG and anti-ZNF274 mouse IgG. The signal from the mouse anti-ZNF274 IgG is indicated.
