# Supplementary material for: ZNF274 Recruits the Histone Methyltransferase SETDB1 to the 3′ Ends of ZNF Genes
Source: PLoS One. 2010 Dec 8;5(12):e15082. doi: 10.1371/journal.pone.0015082 (PMC2999557; doi:10.1371/journal.pone.0015082)
Supplement: Figure S3 — qPCR analysis of ZNF274 ChIP-seq libraries prepared from two different fractions. ZNF274 libraries were prepared as described in the Materials and Methods section. Following 14 cycles of PCR amplification and agarose gel electrophoresis, two different sized fractions (200-400 bp and 400-600 bp) were excised and extracted and compared to a library of input non-ChIP enriched DNA. The targets analyzed are shown below and primer sequences are listed in the supplementary information. (PDF) [file pone.0015082.s003.pdf]

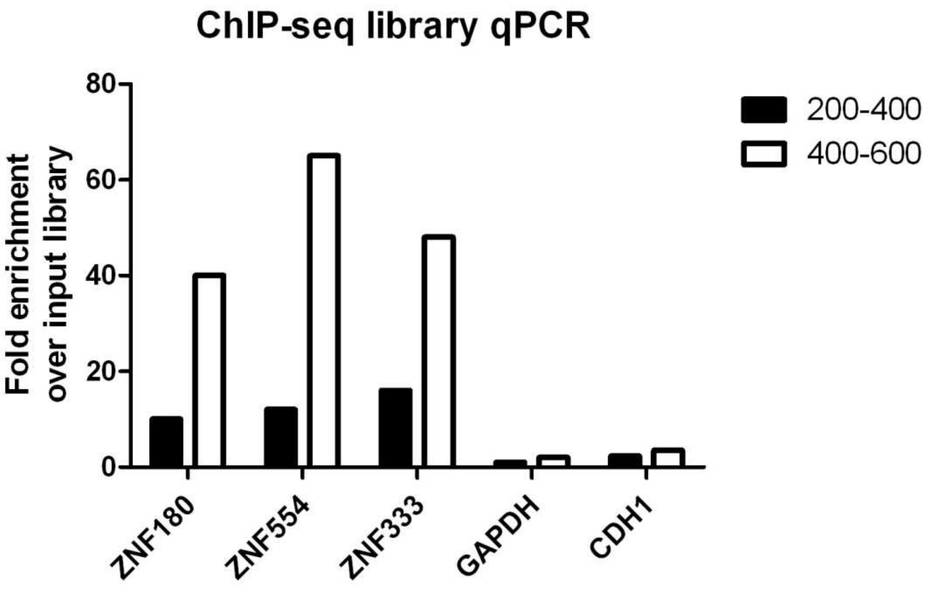

Frietze_Figure S3. qPCR analysis of ZNF274 ChIP-seq libraries prepared from two different fractions. ZNF274 libraries were prepared as described in the Materials and Methods section. Following 14 cycles of PCR amplification and agarose gel electrophoresis, two different sized fractions (200-400 bp and 400-600 bp) were excised and extracted and compared to a library of input non-ChIP enriched DNA. The targets analyzed are shown below and primer sequences are listed in the supplementary Information.
