# Supplementary material for: ZNF274 Recruits the Histone Methyltransferase SETDB1 to the 3′ Ends of ZNF Genes
Source: PLoS One. 2010 Dec 8;5(12):e15082. doi: 10.1371/journal.pone.0015082 (PMC2999557; doi:10.1371/journal.pone.0015082)
Supplement: Figure S4 — Enriched motifs found in ZNF274 binding sites. W-ChIPMotifs was used to extract enriched motifs present in ZNF274 binding sites as described in the Materials and Methods section. Shown are the detected motifs with their SeqLOGOs, PWMs, core and PWM scores, P-values and Bonferroni correction P-value at different percentile levels for each identified motif. (PDF) [file pone.0015082.s004.pdf]

ChIPMotifs Result

Motif Name : ZNF274W1

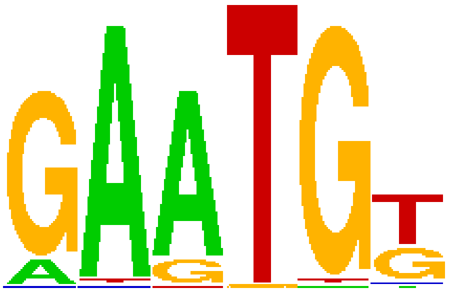

Confidence_Level	Motif_Name	Core_Score	PWM_Score	P-Value	Bonferroni Corrected P-value
99.9%	ZNF274W1	1.000	1.000	4.8571138735125e-208	1.63684737537371e-205
99.5%	ZNF274W1	1.000	1.000	4.8571138735125e-208	1.63684737537371e-205
99.0%	ZNF274W1	1.000	1.000	4.8571138735125e-208	1.63684737537371e-205

ID V$ZNF274W1
MATR_LENGTH 6
CORE_START 1
CORE_LENGTH 5
CORE_MAXIMAL 4304
MAXIMAL 4819
FREQ_T 0.266631280962491
FREQ_A 0.330856334041047
FREQ_C 0.0159235668789809
FREQ_G 0.386588818117481
1 A:126 C:23 G:793 T:0
2 A:900 C:19 G:0 T:23
3 A:793 C:0 G:125 T:24
4 A:0 C:0 G:23 T:919
5 A:17 C:0 G:899 T:26
6 A:34 C:48 G:345 T:515

Motif Name : ZNF274W12

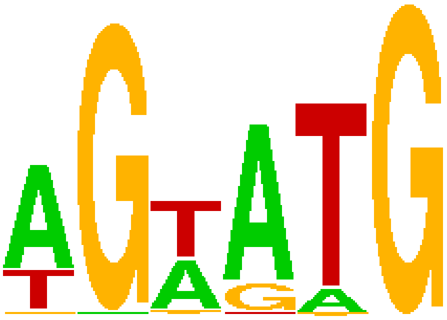

Confidence_Level	Motif_Name	Core_Score	PWM_Score	P-Value	Bonferroni Corrected P-value
99.9%	ZNF274W12	1.000	1.000	5.27176917766827e-149	1.77658621287421e-146
99.5%	ZNF274W12	1.000	1.000	5.27176917766827e-149	1.77658621287421e-146
99.0%	ZNF274W12	1.000	1.000	5.27176917766827e-149	1.77658621287421e-146

ID V$ZNF274W12
MATR_LENGTH 6
CORE_START 2
CORE_LENGTH 5
CORE_MAXIMAL 3061
MAXIMAL 3571
FREQ_T 0.277853569804457
FREQ_A 0.34924965893588
FREQ_C 0.0034106412005457
FREQ_G 0.369486130059118
1 A:510 C:0 G:23 T:200
2 A:12 C:0 G:721 T:0
3 A:326 C:15 G:19 T:373
4 A:600 C:0 G:118 T:15
5 A:88 C:0 G:11 T:634
6 A:0 C:0 G:733 T:0

Motif Name : ZNF274W16

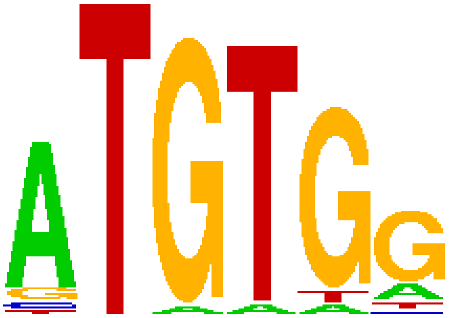

Confidence_Level	Motif_Name	Core_Score	PWM_Score	P-Value	Bonferroni Corrected P-value
99.9%	ZNF274W16	1.000	1.000	1.20629345993044e-206	4.06520895996558e-204
99.5%	ZNF274W16	1.000	1.000	1.20629345993044e-206	4.06520895996558e-204
99.0%	ZNF274W16	1.000	1.000	1.20629345993044e-206	4.06520895996558e-204

ID V$ZNF274W16
MATR_LENGTH 6
CORE_START 1
CORE_LENGTH 5
CORE_MAXIMAL 2414
MAXIMAL 2775
FREQ_T 0.356046065259117
FREQ_A 0.192258477287268
FREQ_C 0.0159948816378759
FREQ_G 0.435700575815739
1 A:438 C:23 G:42 T:18
2 A:0 C:0 G:0 T:521
3 A:19 C:0 G:502 T:0
4 A:25 C:0 G:0 T:496
5 A:29 C:0 G:457 T:35
6 A:90 C:27 G:361 T:43

Motif Name : ZNF274W5

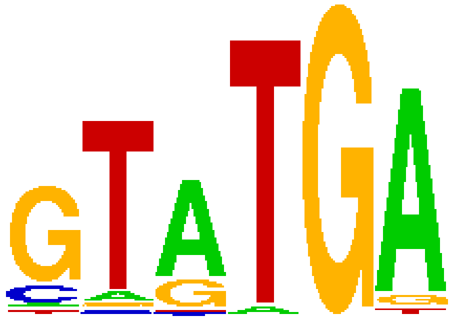

Confidence_Level	Motif_Name	Core_Score	PWM_Score	P-Value	Bonferroni Corrected P-value
99.9%	ZNF274W5	1.000	1.000	3.56275791691297e-161	1.20064941799967e-158
99.5%	ZNF274W5	1.000	1.000	3.56275791691297e-161	1.20064941799967e-158
99.0%	ZNF274W5	1.000	1.000	3.56275791691297e-161	1.20064941799967e-158

ID V$ZNF274W5
MATR_LENGTH 6
CORE_START 2
CORE_LENGTH 5
CORE_MAXIMAL 2541
MAXIMAL 2968
FREQ_T 0.325744308231173
FREQ_A 0.294220665499124
FREQ_C 0.0350262697022767
FREQ_G 0.345008756567426
1 A:27 C:90 G:427 T:27
2 A:34 C:18 G:21 T:498
3 A:407 C:12 G:132 T:20
4 A:23 C:0 G:0 T:548
5 A:0 C:0 G:571 T:0
6 A:517 C:0 G:31 T:23

Motif Name : ZNF274W9

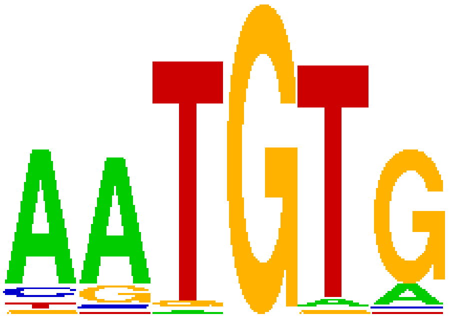

Confidence_Level	Motif_Name	Core_Score	PWM_Score	P-Value	Bonferroni Corrected P-value
99.9%	ZNF274W9	1.000	1.000	2.73198081710543e-208	9.2067753536453e-206
99.5%	ZNF274W9	1.000	1.000	2.73198081710543e-208	9.2067753536453e-206
99.0%	ZNF274W9	1.000	1.000	2.73198081710543e-208	9.2067753536453e-206

ID V$ZNF274W9
MATR_LENGTH 6
CORE_START 2
CORE_LENGTH 5
CORE_MAXIMAL 3031
MAXIMAL 3586
FREQ_T 0.332098765432099
FREQ_A 0.303703703703704
FREQ_C 0.0251851851851852
FREQ_G 0.339012345679012
1 A:555 C:55 G:28 T:37
2 A:541 C:28 G:86 T:20
3 A:17 C:0 G:21 T:637
4 A:0 C:0 G:675 T:0
5 A:26 C:0 G:17 T:632
6 A:91 C:19 G:546 T:19

Motif Name : ZNF274W13

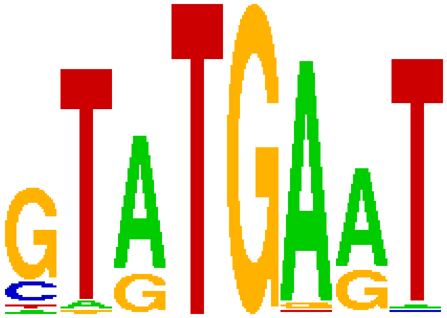

Confidence_Level	Motif_Name	Core_Score	PWM_Score	P-Value	Bonferroni Corrected P-value
99.9%	ZNF274W13	1.000	1.000	1.47535319177974e-104	4.97194025629772e-102
99.5%	ZNF274W13	1.000	0.948	2.51491223770939e-146	8.47525424108064e-144
99.0%	ZNF274W13	1.000	0.920	2.01321419464735e-184	6.78453183596157e-182

ID V$ZNF274W13
MATR_LENGTH 8
CORE_START 2
CORE_LENGTH 5
CORE_MAXIMAL 1469
MAXIMAL 2216
FREQ_T 0.3698738170347
FREQ_A 0.310725552050473
FREQ_C 0.027602523659306
FREQ_G 0.291798107255521
1 A:14 C:55 G:233 T:15
2 A:14 C:2 G:4 T:297
3 A:238 C:2 G:77 T:0
4 A:0 C:0 G:0 T:317
5 A:0 C:0 G:317 T:0
6 A:300 C:1 G:10 T:6
7 A:213 C:5 G:97 T:2
8 A:9 C:5 G:2 T:301

Motif Name : ZNF274W17

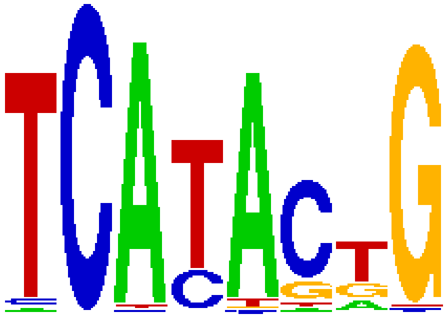

Confidence_Level	Motif_Name	Core_Score	PWM_Score	P-Value	Bonferroni Corrected P-value
99.9%	ZNF274W17	1.000	1.000	1.05235496876334e-120	3.54643624473246e-118
99.5%	ZNF274W17	1.000	0.940	2.64328265641424e-146	8.90786255211599e-144
99.0%	ZNF274W17	1.000	0.914	1.39807277288523e-152	4.71150524462323e-150

ID V$ZNF274W17
MATR_LENGTH 8
CORE_START 1
CORE_LENGTH 5
CORE_MAXIMAL 1624
MAXIMAL 2437
FREQ_T 0.297887323943662
FREQ_A 0.26830985915493
FREQ_C 0.260915492957746
FREQ_G 0.172887323943662
1 A:9 C:13 G:2 T:331
2 A:0 C:354 G:0 T:1
3 A:342 C:5 G:0 T:8
4 A:4 C:85 G:0 T:266
5 A:331 C:4 G:6 T:14
6 A:15 C:266 G:57 T:17
7 A:58 C:10 G:83 T:204
8 A:3 C:4 G:343 T:5

Motif Name : ZNF274W2

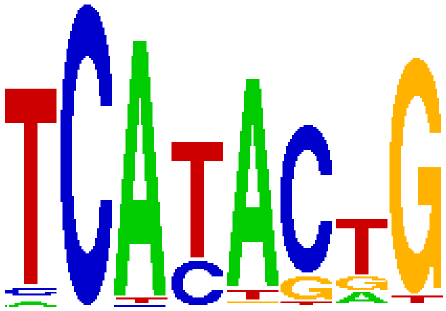

Confidence_Level	Motif_Name	Core_Score	PWM_Score	P-Value	Bonferroni Corrected P-value
99.9%	ZNF274W2	1.000	1.000	1.05235496876334e-120	3.54643624473246e-118
99.5%	ZNF274W2	1.000	0.930	2.64328265641424e-146	8.90786255211599e-144
99.0%	ZNF274W2	1.000	0.902	1.39807277288523e-152	4.71150524462323e-150

ID V$ZNF274W2
MATR_LENGTH 8
CORE_START 1
CORE_LENGTH 5
CORE_MAXIMAL 1619
MAXIMAL 2488
FREQ_T 0.300414364640884
FREQ_A 0.261395027624309
FREQ_C 0.276588397790055
FREQ_G 0.161602209944751
1 A:15 C:19 G:2 T:326
2 A:0 C:360 G:1 T:1
3 A:347 C:5 G:0 T:10
4 A:3 C:104 G:0 T:255
5 A:331 C:4 G:8 T:19
6 A:1 C:296 G:53 T:12
7 A:57 C:10 G:63 T:232
8 A:3 C:3 G:341 T:15

Motif Name : ZNF274W6

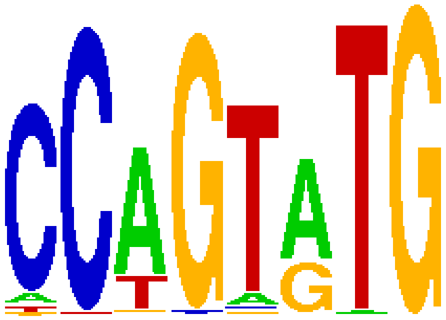

Confidence_Level	Motif_Name	Core_Score	PWM_Score	P-Value	Bonferroni Corrected P-value
99.9%	ZNF274W6	1.000	1.000	2.91996415798946e-128	9.84027921242448e-126
99.5%	ZNF274W6	1.000	0.896	6.06398067979717e-156	2.04356148909165e-153
99.0%	ZNF274W6	1.000	0.882	4.58986972475418e-157	1.54678609724216e-154

ID V$ZNF274W6
MATR_LENGTH 8
CORE_START 4
CORE_LENGTH 5
CORE_MAXIMAL 1192
MAXIMAL 1891
FREQ_T 0.268396226415094
FREQ_A 0.194811320754717
FREQ_C 0.239150943396226
FREQ_G 0.297641509433962
1 A:14 C:237 G:4 T:10
2 A:1 C:260 G:0 T:4
3 A:202 C:1 G:6 T:56
4 A:2 C:3 G:259 T:1
5 A:17 C:6 G:6 T:236
6 A:172 C:0 G:91 T:2
7 A:5 C:0 G:0 T:260
8 A:0 C:0 G:265 T:0

Motif Name : ZNF274W10

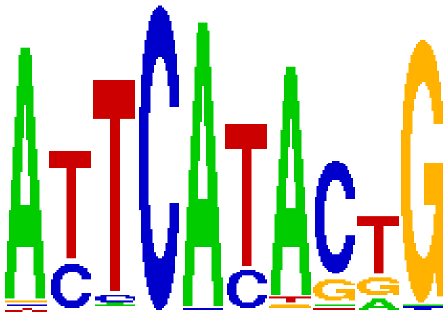

Confidence_Level	Motif_Name	Core_Score	PWM_Score	P-Value	Bonferroni Corrected P-value
99.9%	ZNF274W10	1.000	0.929	6.57186211201308e-129	2.21471753174841e-126
99.5%	ZNF274W10	1.000	0.889	9.77481647293076e-145	3.29411315137767e-142
99.0%	ZNF274W10	1.000	0.852	6.83490553314706e-192	2.30336316467056e-189

ID V$ZNF274W10
MATR_LENGTH 10
CORE_START 3
CORE_LENGTH 5
CORE_MAXIMAL 1313
MAXIMAL 2454
FREQ_T 0.308070175438596
FREQ_A 0.310175438596491
FREQ_C 0.239649122807018
FREQ_G 0.142105263157895
1 A:273 C:4 G:5 T:3
2 A:2 C:87 G:1 T:195
3 A:8 C:12 G:3 T:262
4 A:0 C:284 G:1 T:0
5 A:280 C:4 G:0 T:1
6 A:0 C:64 G:1 T:220
7 A:267 C:2 G:7 T:9
8 A:10 C:219 G:48 T:8
9 A:40 C:4 G:63 T:178
10 A:4 C:3 G:276 T:2

Motif Name : ZNF274W14

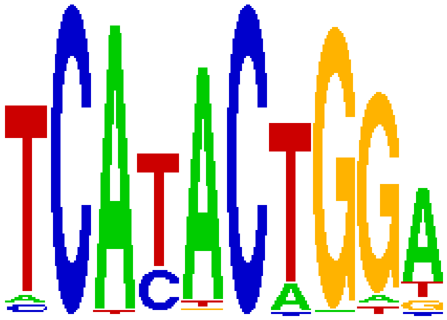

Confidence_Level	Motif_Name	Core_Score	PWM_Score	P-Value	Bonferroni Corrected P-value
99.9%	ZNF274W14	1.000	0.933	1.02709657304284e-84	3.46131545115437e-82
99.5%	ZNF274W14	1.000	0.853	4.70476342280322e-152	1.58550527348469e-149
99.0%	ZNF274W14	1.000	0.828	1.90541194826958e-171	6.42123826566848e-169

ID V$ZNF274W14
MATR_LENGTH 10
CORE_START 2
CORE_LENGTH 5
CORE_MAXIMAL 925
MAXIMAL 1794
FREQ_T 0.2665
FREQ_A 0.2935
FREQ_C 0.2385
FREQ_G 0.2015
1 A:10 C:10 G:2 T:178
2 A:0 C:200 G:0 T:0
3 A:196 C:0 G:0 T:4
4 A:3 C:56 G:0 T:141
5 A:188 C:2 G:3 T:7
6 A:0 C:200 G:0 T:0
7 A:30 C:3 G:1 T:166
8 A:3 C:0 G:196 T:1
9 A:9 C:1 G:181 T:9
10 A:148 C:5 G:20 T:27

Motif Name : ZNF274W18

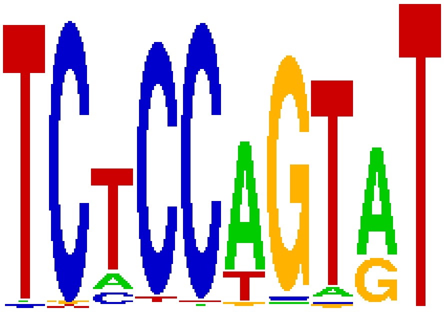

Confidence_Level	Motif_Name	Core_Score	PWM_Score	P-Value	Bonferroni Corrected P-value
99.9%	ZNF274W18	1.000	0.935	3.82639593475685e-132	1.28949543001306e-129
99.5%	ZNF274W18	1.000	0.897	9.20716516326833e-156	3.10281466002143e-153
99.0%	ZNF274W18	1.000	0.846	4.35070912083292e-170	1.46618897372069e-167

ID V$ZNF274W18
MATR_LENGTH 10
CORE_START 1
CORE_LENGTH 5
CORE_MAXIMAL 998
MAXIMAL 1925
FREQ_T 0.388837209302326
FREQ_A 0.168837209302326
FREQ_C 0.311627906976744
FREQ_G 0.130697674418605
1 A:2 C:2 G:1 T:210
2 A:0 C:211 G:2 T:2
3 A:28 C:24 G:2 T:161
4 A:1 C:206 G:1 T:7
5 A:2 C:210 G:0 T:3
6 A:167 C:3 G:4 T:41
7 A:4 C:6 G:203 T:2
8 A:11 C:5 G:3 T:196
9 A:147 C:3 G:65 T:0
10 A:1 C:0 G:0 T:214

Motif Name : ZNF274W3

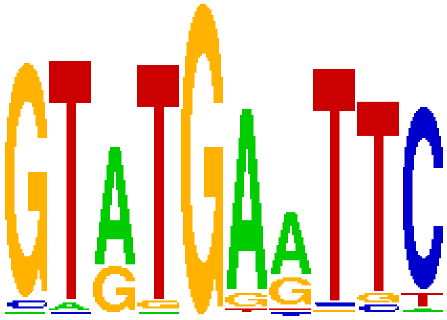

Confidence_Level	Motif_Name	Core_Score	PWM_Score	P-Value	Bonferroni Corrected P-value
99.9%	ZNF274W3	1.000	0.935	4.88965029153297e-110	1.64781214824661e-107
99.5%	ZNF274W3	1.000	0.898	6.49122600458787e-152	2.18754316354611e-149
99.0%	ZNF274W3	1.000	0.843	2.08539846242345e-158	7.02779281836703e-156

ID V$ZNF274W3
MATR_LENGTH 10
CORE_START 1
CORE_LENGTH 5
CORE_MAXIMAL 1080
MAXIMAL 2086
FREQ_T 0.38744769874477
FREQ_A 0.232217573221757
FREQ_C 0.106276150627615
FREQ_G 0.274058577405858
1 A:5 C:10 G:224 T:0
2 A:10 C:4 G:0 T:225
3 A:168 C:0 G:70 T:1
4 A:5 C:0 G:10 T:224
5 A:0 C:0 G:239 T:0
6 A:210 C:2 G:20 T:7
7 A:147 C:8 G:72 T:12
8 A:2 C:7 G:5 T:225
9 A:1 C:12 G:13 T:213
10 A:7 C:211 G:2 T:19

Motif Name : ZNF274W7

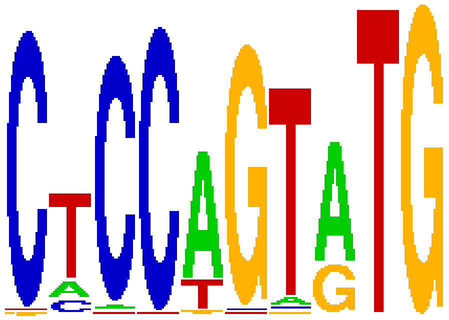

Confidence_Level	Motif_Name	Core_Score	PWM_Score	P-Value	Bonferroni Corrected P-value
99.9%	ZNF274W7	1.000	0.896	1.09883113615416e-135	3.70306092883952e-133
99.5%	ZNF274W7	1.000	0.870	7.7279159349673e-157	2.60430767008398e-154
99.0%	ZNF274W7	1.000	0.831	1.65527487619487e-157	5.57827633277671e-155

ID V$ZNF274W7
MATR_LENGTH 10
CORE_START 6
CORE_LENGTH 5
CORE_MAXIMAL 974
MAXIMAL 1911
FREQ_T 0.287323943661972
FREQ_A 0.169953051643192
FREQ_C 0.308920187793427
FREQ_G 0.233802816901408
1 A:0 C:208 G:2 T:3
2 A:31 C:27 G:3 T:152
3 A:5 C:203 G:1 T:4
4 A:1 C:209 G:0 T:3
5 A:165 C:3 G:4 T:41
6 A:2 C:3 G:206 T:2
7 A:9 C:5 G:4 T:195
8 A:148 C:0 G:65 T:0
9 A:1 C:0 G:0 T:212
10 A:0 C:0 G:213 T:0

Motif Name : ZNF274W11

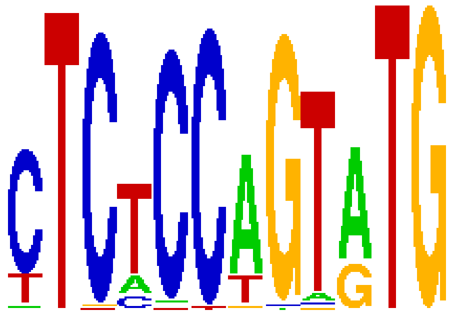

Confidence_Level	Motif_Name	Core_Score	PWM_Score	P-Value	Bonferroni Corrected P-value
99.9%	ZNF274W11	1.000	0.856	1.30282649794947e-150	4.39052529808971e-148
99.5%	ZNF274W11	1.000	0.838	1.09790337689357e-158	3.69993438013133e-156
99.0%	ZNF274W11	1.000	0.802	4.07819046193821e-163	1.37435018567318e-160

ID V$ZNF274W11
MATR_LENGTH 12
CORE_START 8
CORE_LENGTH 5
CORE_MAXIMAL 944
MAXIMAL 2219
FREQ_T 0.337359098228663
FREQ_A 0.143719806763285
FREQ_C 0.32085346215781
FREQ_G 0.198067632850242
1 A:5 C:160 G:3 T:39
2 A:1 C:0 G:1 T:205
3 A:0 C:201 G:3 T:3
4 A:31 C:23 G:3 T:150
5 A:4 C:198 G:1 T:4
6 A:1 C:202 G:0 T:4
7 A:159 C:3 G:7 T:38
8 A:2 C:3 G:201 T:1
9 A:9 C:5 G:5 T:188
10 A:143 C:2 G:62 T:0
11 A:1 C:0 G:0 T:206
12 A:1 C:0 G:206 T:0

Motif Name : ZNF274W15

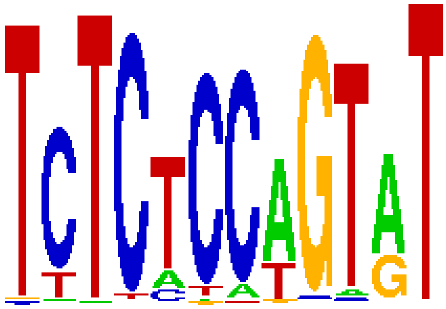

Confidence_Level	Motif_Name	Core_Score	PWM_Score	P-Value	Bonferroni Corrected P-value
99.9%	ZNF274W15	1.000	0.904	7.22036635342417e-146	2.43326346110395e-143
99.5%	ZNF274W15	1.000	0.838	8.9960856096223e-166	3.03168085044272e-163
99.0%	ZNF274W15	1.000	0.808	6.35690489325428e-167	2.14227694902669e-164

ID V$ZNF274W15
MATR_LENGTH 12
CORE_START 3
CORE_LENGTH 5
CORE_MAXIMAL 976
MAXIMAL 2276
FREQ_T 0.431782945736434
FREQ_A 0.14031007751938
FREQ_C 0.314341085271318
FREQ_G 0.113565891472868
1 A:0 C:2 G:4 T:209
2 A:5 C:174 G:2 T:34
3 A:3 C:0 G:1 T:211
4 A:0 C:207 G:2 T:6
5 A:28 C:20 G:2 T:165
6 A:3 C:197 G:3 T:12
7 A:15 C:196 G:0 T:4
8 A:152 C:3 G:4 T:56
9 A:2 C:5 G:207 T:1
10 A:8 C:5 G:2 T:200
11 A:145 C:1 G:66 T:3
12 A:1 C:1 G:0 T:213

Motif Name : ZNF274W19

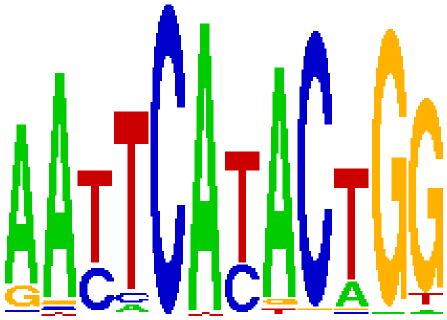

Confidence_Level	Motif_Name	Core_Score	PWM_Score	P-Value	Bonferroni Corrected P-value
99.9%	ZNF274W19	1.000	0.861	4.70476342280322e-152	1.58550527348469e-149
99.5%	ZNF274W19	1.000	0.833	5.28116093050507e-165	1.77975123358021e-162
99.0%	ZNF274W19	1.000	0.805	2.1274955519829e-169	7.16966001018237e-167

ID V$ZNF274W19
MATR_LENGTH 12
CORE_START 5
CORE_LENGTH 5
CORE_MAXIMAL 980
MAXIMAL 2251
FREQ_T 0.259345794392523
FREQ_A 0.332554517133956
FREQ_C 0.230529595015576
FREQ_G 0.177570093457944
1 A:180 C:5 G:27 T:2
2 A:200 C:5 G:6 T:3
3 A:3 C:70 G:2 T:139
4 A:10 C:14 G:2 T:188
5 A:0 C:214 G:0 T:0
6 A:211 C:1 G:0 T:2
7 A:0 C:67 G:1 T:146
8 A:200 C:0 G:7 T:7
9 A:1 C:209 G:3 T:1
10 A:38 C:6 G:7 T:163
11 A:4 C:0 G:209 T:1
12 A:7 C:1 G:192 T:14

Motif Name : ZNF274W4

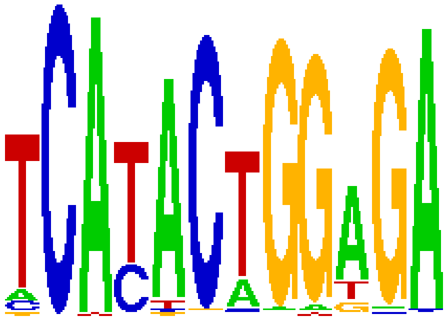

Confidence_Level	Motif_Name	Core_Score	PWM_Score	P-Value	Bonferroni Corrected P-value
99.9%	ZNF274W4	1.000	0.879	1.10070849739946e-145	3.70938763623618e-143
99.5%	ZNF274W4	1.000	0.823	2.00728336188443e-163	6.76454492955053e-161
99.0%	ZNF274W4	1.000	0.789	2.32173680918721e-167	7.8242530469609e-165

ID V$ZNF274W4
MATR_LENGTH 12
CORE_START 2
CORE_LENGTH 5
CORE_MAXIMAL 933
MAXIMAL 2196
FREQ_T 0.21264367816092
FREQ_A 0.330049261083744
FREQ_C 0.203612479474548
FREQ_G 0.253694581280788
1 A:15 C:13 G:3 T:172
2 A:0 C:203 G:0 T:0
3 A:201 C:0 G:0 T:2
4 A:0 C:60 G:0 T:143
5 A:188 C:3 G:3 T:9
6 A:1 C:198 G:3 T:1
7 A:37 C:8 G:1 T:157
8 A:4 C:1 G:197 T:1
9 A:6 C:1 G:194 T:2
10 A:149 C:3 G:21 T:30
11 A:4 C:4 G:195 T:0
12 A:199 C:2 G:1 T:1

Motif Name : ZNF274W8

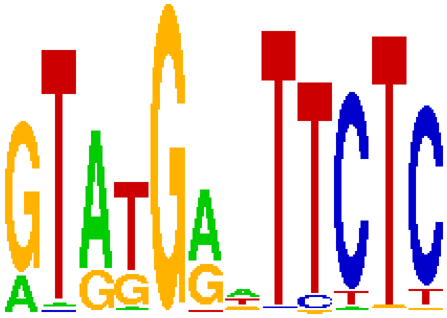

Confidence_Level	Motif_Name	Core_Score	PWM_Score	P-Value	Bonferroni Corrected P-value
99.9%	ZNF274W8	1.000	0.889	1.08119021728668e-196	3.64361103225611e-194
99.5%	ZNF274W8	1.000	0.848	2.60117986193699e-236	8.76597613472766e-234
99.0%	ZNF274W8	1.000	0.823	7.51455566755363e-255	2.53240525996557e-252

ID V$ZNF274W8
MATR_LENGTH 12
CORE_START 8
CORE_LENGTH 5
CORE_MAXIMAL 1604
MAXIMAL 3386
FREQ_T 0.417630057803468
FREQ_A 0.170279383429672
FREQ_C 0.165221579961464
FREQ_G 0.246868978805395
1 A:68 C:2 G:276 T:0
2 A:9 C:7 G:0 T:330
3 A:263 C:1 G:82 T:0
4 A:18 C:2 G:90 T:236
5 A:0 C:1 G:345 T:0
6 A:203 C:1 G:127 T:15
7 A:129 C:28 G:82 T:107
8 A:1 C:5 G:2 T:338
9 A:2 C:24 G:4 T:316
10 A:9 C:310 G:1 T:26
11 A:2 C:1 G:7 T:336
12 A:3 C:304 G:9 T:30

Motif Name : ZNF274F1

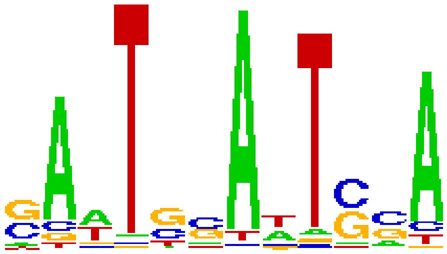

Confidence_Level	Motif_Name	Core_Score	PWM_Score	P-Value	Bonferroni Corrected P-value
99.9%	ZNF274F1	1.000	0.863	3.27780202633893e-20	1.10461928287622e-17
99.5%	ZNF274F1	1.000	0.786	1.43747933567108e-37	4.84430536121154e-35
99.0%	ZNF274F1	1.000	0.736	9.66519000364622e-46	3.25716903122878e-43

ID V$ZNF274F1
MATR_LENGTH 12
CORE_START 3
CORE_LENGTH 5
CORE_MAXIMAL 1079
MAXIMAL 2518
FREQ_T 0.269535113748764
FREQ_A 0.333580613254204
FREQ_C 0.201038575667656
FREQ_G 0.195845697329377
1 A:44 C:127 G:146 T:20
2 A:272 C:27 G:21 T:17
3 A:143 C:31 G:39 T:124
4 A:7 C:7 G:7 T:316
5 A:22 C:94 G:163 T:58
6 A:41 C:145 G:114 T:37
7 A:312 C:6 G:3 T:16
8 A:121 C:48 G:33 T:135
9 A:14 C:9 G:10 T:304
10 A:22 C:151 G:144 T:20
11 A:66 C:146 G:104 T:21
12 A:285 C:22 G:8 T:22

Motif Name : ZNF274F11

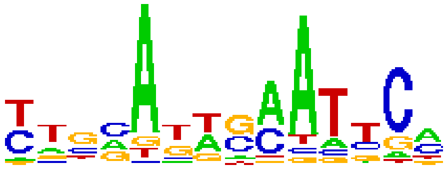

Confidence_Level	Motif_Name	Core_Score	PWM_Score	P-Value	Bonferroni Corrected P-value
99.9%	ZNF274F11	1.000	0.905	3.3838636827013e-49	1.14036206107034e-46
99.5%	ZNF274F11	1.000	0.853	1.39943822328099e-118	4.71610681245694e-116
99.0%	ZNF274F11	1.000	0.827	2.30194805588884e-136	7.75756494834539e-134

ID V$ZNF274F11
MATR_LENGTH 14
CORE_START 9
CORE_LENGTH 5
CORE_MAXIMAL 1069
MAXIMAL 2518
FREQ_T 0.261763459092836
FREQ_A 0.301186943620178
FREQ_C 0.248622297583722
FREQ_G 0.188427299703264
1 A:32 C:139 G:16 T:150
2 A:79 C:31 G:59 T:168
3 A:26 C:93 G:146 T:72
4 A:100 C:135 G:91 T:11
5 A:274 C:9 G:27 T:27
6 A:36 C:38 G:111 T:152
7 A:128 C:9 G:67 T:133
8 A:47 C:121 G:151 T:18
9 A:186 C:115 G:17 T:19
10 A:270 C:21 G:21 T:25
11 A:50 C:38 G:33 T:216
12 A:23 C:78 G:67 T:169
13 A:30 C:228 G:59 T:20
14 A:140 C:118 G:24 T:55

Motif Name : ZNF274M1

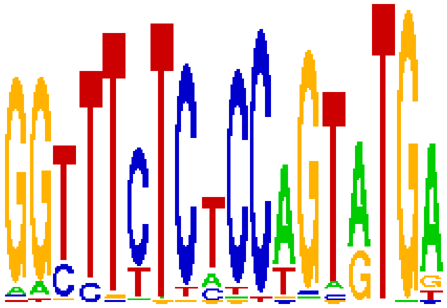

Confidence_Level	Motif_Name	Core_Score	PWM_Score	P-Value	Bonferroni Corrected P-value
99.9%	ZNF274M1	1.000	0.785	1.21930911120763e-156	4.10907170476971e-154
99.5%	ZNF274M1	1.000	0.745	8.58069727650911e-160	2.89169498218357e-157
99.0%	ZNF274M1	1.000	0.734	8.58069727650911e-160	2.89169498218357e-157

ID V$ZNF274M1
MATR_LENGTH 18
CORE_START 13
CORE_LENGTH 5
CORE_MAXIMAL 941
MAXIMAL 3292
FREQ_T 0.378306878306878
FREQ_A 0.146031746031746
FREQ_C 0.233068783068783
FREQ_G 0.242592592592593
1 A:10 C:4 G:192 T:4
2 A:14 C:0 G:190 T:6
3 A:1 C:49 G:5 T:155
4 A:1 C:21 G:0 T:188
5 A:0 C:3 G:5 T:202
6 A:6 C:160 G:3 T:41
7 A:2 C:1 G:2 T:205
8 A:1 C:195 G:4 T:10
9 A:33 C:27 G:6 T:144
10 A:4 C:194 G:3 T:9
11 A:0 C:203 G:1 T:6
12 A:153 C:5 G:7 T:45
13 A:4 C:5 G:199 T:2
14 A:10 C:9 G:3 T:188
15 A:139 C:0 G:70 T:1
16 A:1 C:1 G:0 T:208
17 A:3 C:0 G:207 T:0
18 A:170 C:4 G:20 T:16

Motif Name : ZNF274M2

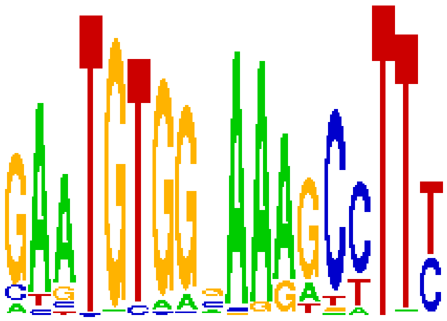

Confidence_Level	Motif_Name	Core_Score	PWM_Score	P-Value	Bonferroni Corrected P-value
99.9%	ZNF274M2	1.000	0.776	1.5214616407077e-243	5.12732572918495e-241
99.5%	ZNF274M2	1.000	0.747	4.7380172072652e-244	1.59671179884837e-241
99.0%	ZNF274M2	1.000	0.718	3.75049655966161e-247	1.26391734060596e-244

ID V$ZNF274M2
MATR_LENGTH 18
CORE_START 4
CORE_LENGTH 5
CORE_MAXIMAL 1005
MAXIMAL 3228
FREQ_T 0.280907668231612
FREQ_A 0.283255086071987
FREQ_C 0.149452269170579
FREQ_G 0.286384976525822
1 A:16 C:23 G:172 T:2
2 A:190 C:8 G:1 T:14
3 A:166 C:13 G:24 T:10
4 A:0 C:2 G:0 T:211
5 A:4 C:1 G:207 T:1
6 A:0 C:11 G:1 T:201
7 A:11 C:3 G:197 T:2
8 A:17 C:5 G:189 T:2
9 A:49 C:66 G:83 T:15
10 A:204 C:5 G:4 T:0
11 A:201 C:2 G:10 T:0
12 A:170 C:1 G:40 T:2
13 A:32 C:3 G:160 T:18

14 A:4 C:189 G:7 T:13
15 A:17 C:149 G:0 T:47
16 A:0 C:0 G:0 T:213
17 A:3 C:1 G:1 T:208
18 A:2 C:91 G:2 T:118

Motif Name : ZNF274M3

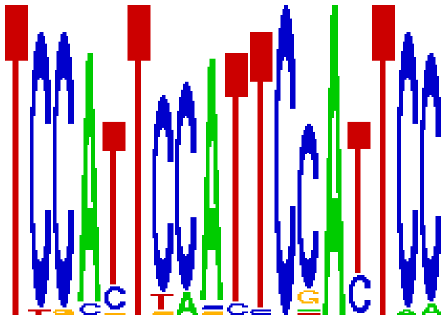

Confidence_Level	Motif_Name	Core_Score	PWM_Score	P-Value	Bonferroni Corrected P-value
99.9%	ZNF274M3	1.000	0.833	2.08538758926456e-63	7.02775617582157e-61
99.5%	ZNF274M3	1.000	0.780	1.09041110878462e-63	3.67468543660417e-61
99.0%	ZNF274M3	1.000	0.740	2.4823196775401e-61	8.36541731331014e-59

ID V$ZNF274M3
MATR_LENGTH 18
CORE_START 14
CORE_LENGTH 5
CORE_MAXIMAL 174
MAXIMAL 627
FREQ_T 0.370870870870871
FREQ_A 0.172672672672673
FREQ_C 0.445945945945946
FREQ_G 0.0105105105105105
1 A:0 C:0 G:0 T:37
2 A:0 C:36 G:0 T:1
3 A:0 C:36 G:1 T:0
4 A:35 C:2 G:0 T:0
5 A:0 C:5 G:1 T:31
6 A:0 C:0 G:0 T:37
7 A:0 C:33 G:1 T:3
8 A:4 C:33 G:0 T:0

9 A:35 C:1 G:1 T:0
10 A:0 C:2 G:0 T:35
11 A:0 C:1 G:0 T:36
12 A:0 C:37 G:0 T:0
13 A:1 C:32 G:3 T:1
14 A:37 C:0 G:0 T:0
15 A:0 C:8 G:0 T:29
16 A:0 C:0 G:0 T:37
17 A:1 C:36 G:0 T:0
18 A:2 C:35 G:0 T:0

Motif Name : ZNF274M12

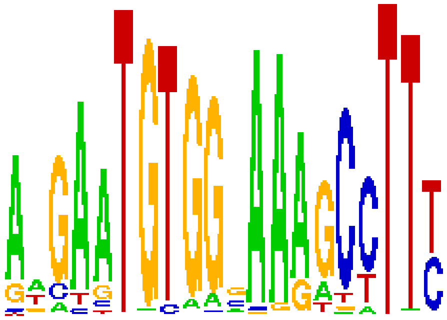

Confidence_Level	Motif_Name	Core_Score	PWM_Score	P-Value	Bonferroni Corrected P-value
99.9%	ZNF274M12	1.000	0.769	1.9230603507472e-240	6.48071338201806e-238
99.5%	ZNF274M12	1.000	0.737	4.46941938693351e-245	1.50619433339659e-242
99.0%	ZNF274M12	1.000	0.723	4.7380172072652e-244	1.59671179884837e-241

ID V$ZNF274M12
MATR_LENGTH 20
CORE_START 6
CORE_LENGTH 5
CORE_MAXIMAL 982
MAXIMAL 3387
FREQ_T 0.271980676328502
FREQ_A 0.314009661835749
FREQ_C 0.139613526570048
FREQ_G 0.274396135265701
1 A:164 C:9 G:30 T:4
2 A:79 C:13 G:41 T:74
3 A:16 C:23 G:166 T:2

4 A:184 C:8 G:1 T:14
5 A:163 C:12 G:23 T:9
6 A:0 C:1 G:0 T:206
7 A:4 C:1 G:201 T:1
8 A:0 C:10 G:0 T:197
9 A:11 C:2 G:192 T:2
10 A:15 C:4 G:186 T:2
11 A:48 C:63 G:81 T:15
12 A:198 C:5 G:4 T:0
13 A:197 C:2 G:8 T:0
14 A:165 C:1 G:39 T:2
15 A:32 C:3 G:154 T:18
16 A:4 C:184 G:7 T:12
17 A:15 C:147 G:0 T:45
18 A:0 C:0 G:0 T:207
19 A:3 C:1 G:1 T:202
20 A:2 C:89 G:2 T:114

Motif Name : ZNF274M11

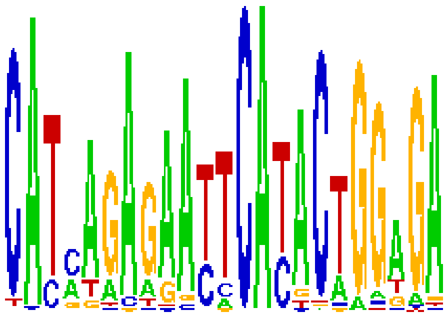

Confidence_Level	Motif_Name	Core_Score	PWM_Score	P-Value	Bonferroni Corrected P-value
99.9%	ZNF274M11	1.000	0.768	1.39807277288523e-152	4.71150524462323e-150
99.5%	ZNF274M11	1.000	0.710	1.65527487619487e-157	5.57827633277671e-155
99.0%	ZNF274M11	1.000	0.699	2.56798675695458e-157	8.65411537093693e-155

ID V$ZNF274M11
MATR_LENGTH 23
CORE_START 13
CORE_LENGTH 5
CORE_MAXIMAL 894
MAXIMAL 3836

FREQ_T 0.188114485470832
FREQ_A 0.394144636224601
FREQ_C 0.202752894909329
FREQ_G 0.214987983395237
1 A:1 C:190 G:1 T:7
2 A:196 C:2 G:1 T:0
3 A:1 C:33 G:1 T:164
4 A:67 C:97 G:29 T:6
5 A:162 C:1 G:13 T:23
6 A:33 C:7 G:151 T:8
7 A:188 C:9 G:2 T:0
8 A:29 C:6 G:148 T:16
9 A:164 C:3 G:28 T:4
10 A:183 C:7 G:8 T:1
11 A:2 C:63 G:2 T:132
12 A:14 C:21 G:4 T:160
13 A:0 C:198 G:0 T:1
14 A:198 C:0 G:1 T:0
15 A:0 C:66 G:0 T:133
16 A:175 C:3 G:11 T:10
17 A:2 C:190 G:3 T:4
18 A:39 C:9 G:5 T:146
19 A:12 C:1 G:186 T:0
20 A:10 C:7 G:178 T:4
21 A:131 C:8 G:26 T:34
22 A:12 C:3 G:181 T:3
23 A:185 C:4 G:5 T:5

Motif Name : ZNF274M13

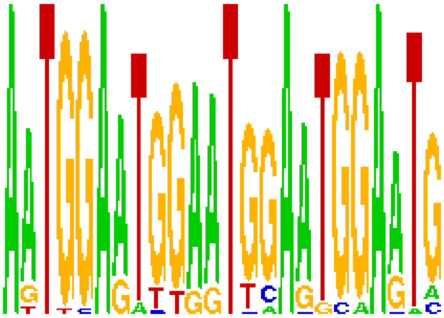

Confidence_Level	Motif_Name	Core_Score	PWM_Score	P-Value	Bonferroni Corrected P-value

99.9%	ZNF274M13	1.000	0.710	4.54051390243522e-67	1.53015318512067e-64
99.5%	ZNF274M13	1.000	0.687	6.72642081740023e-69	2.26680381546388e-66
99.0%	ZNF274M13	1.000	0.677	6.72642081740023e-69	2.26680381546388e-66

ID V$ZNF274M13
MATR_LENGTH 24
CORE_START 3
CORE_LENGTH 5
CORE_MAXIMAL 171
MAXIMAL 789
FREQ_T 0.221064814814815
FREQ_A 0.385416666666667
FREQ_C 0.0162037037037037
FREQ_G 0.377314814814815
1 A:36 C:0 G:0 T:0
2 A:30 C:0 G:4 T:2
3 A:0 C:0 G:0 T:36
4 A:0 C:0 G:35 T:1
5 A:0 C:1 G:35 T:0
6 A:36 C:0 G:0 T:0
7 A:29 C:0 G:7 T:0
8 A:2 C:0 G:0 T:34
9 A:0 C:1 G:31 T:4
10 A:0 C:0 G:32 T:4
11 A:32 C:0 G:4 T:0
12 A:31 C:0 G:5 T:0
13 A:0 C:0 G:0 T:36
14 A:0 C:1 G:30 T:5
15 A:2 C:4 G:30 T:0
16 A:36 C:0 G:0 T:0
17 A:30 C:1 G:5 T:0
18 A:0 C:0 G:2 T:34
19 A:0 C:2 G:34 T:0
20 A:2 C:0 G:34 T:0
21 A:36 C:0 G:0 T:0
22 A:27 C:1 G:8 T:0
23 A:1 C:0 G:0 T:35
24 A:3 C:3 G:30 T:0
