# Supplementary material for: ZNF274 Recruits the Histone Methyltransferase SETDB1 to the 3′ Ends of ZNF Genes
Source: PLoS One. 2010 Dec 8;5(12):e15082. doi: 10.1371/journal.pone.0015082 (PMC2999557; doi:10.1371/journal.pone.0015082)
Supplement: Figure S5 — The similarity of the ZNF274 enriched motifs is compared to the closest known motif for other factors using the STAMP tool. Shown are the matched similar motifs from the STAMP tool. (PDF) [file pone.0015082.s005.pdf]

Frietze_Figure S5

Information from STAMP

The following results were obtained using default options from STAMP

Phylogenetic tree from the Obtained motifs

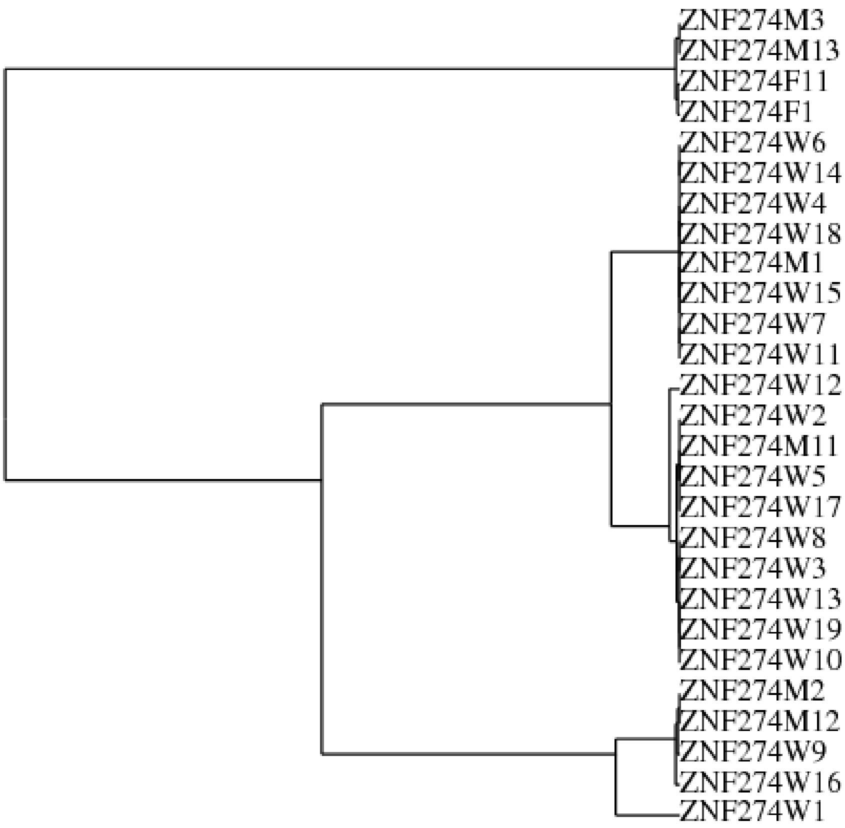

ZNF274W1

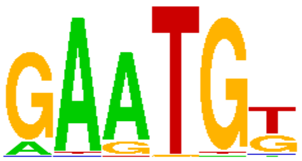

Motif Name	E-Value	Alignment	Weblogo

TEAD	2.0663e-03	-----GAATGK-<br>CNSWGGAATGTR	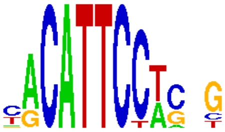
CAT8_Q6	5.0528e-03	----GAATGK<br>GGNNNAATGG	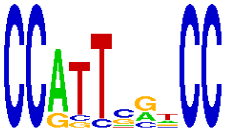
O2_01	8.6408e-03	----------GAATGK<br>YATCTACGTGGAATGA	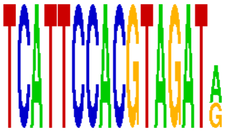
TEF1_Q6	1.1240e-02	MCATTC-<br>-CATYYC	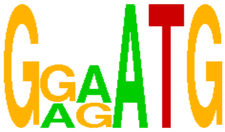
ABAA_01	1.7252e-02	-------GAATGK------<br>NNNNNNGGAATGNRRNNNN	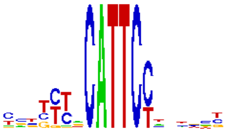

ZNF274W10

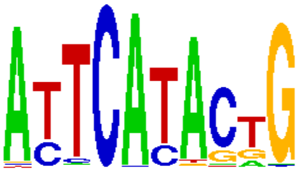

Motif Name	E-Value	Alignment	Weblogo
TBP_01	8.3395e-03	-ATTCATACTG<br>WATTTATA---	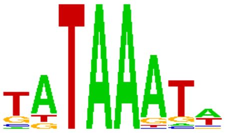
T	2.1352e-02	ATTCATACTG--<br>-TTCACACCTAG	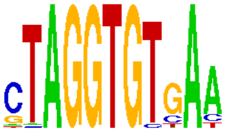
TBP_Q6	2.4921e-02	CAGTATGAAT-<br>--ATATAAANR	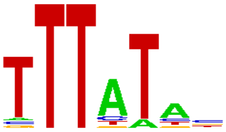

POU1F1_Q6	3.2654e-02	----ATTCATACTG<br>WWTTATNCAT----	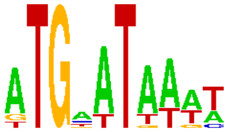
CF2II_01	3.6493e-02	-ATTCATACTG<br>TAYATATAY--	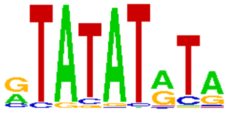

ZNF274W11

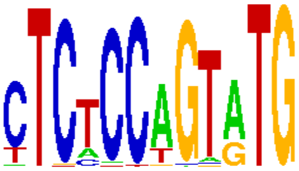

Motif Name	E-Value	Alignment	Weblogo
AREB6_01	1.7979e-02	CATACTGGAGAG--<br>-RNWCAGGTRNRNN	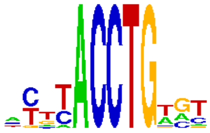
XPF1_Q6	6.3759e-02	-CTCTCCAGTATG<br>GKTSNYCNGA---	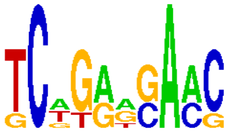
GRH_01	7.9773e-02	CTCTCCAGTATG<br>-AAACCRGT---	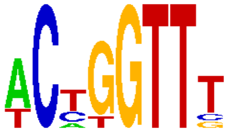
Pax5	8.1162e-02	------CTCTCCAGTATG--<br>YSGYYMCGCWNCRNNNNNCN	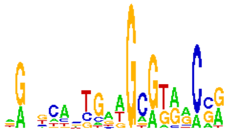
MAT1MC_02	8.4206e-02	-CATACTGGAGAG<br>NRACAATNGR---	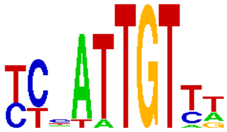

ZNF274W12

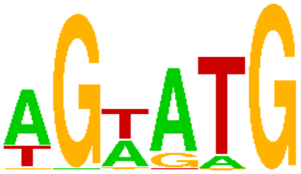

Motif Name	E-Value	Alignment	Weblogo
UNC86_Q2	1.5696e-02	----AGWATG<br>ATTTYKWATG	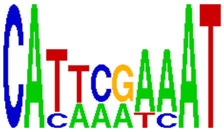
O2_01	3.7207e-02	---------AGWATG-<br>YATCTACGTGGAATGA	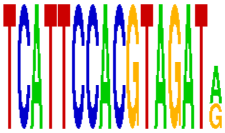
OCT1_05	3.9087e-02	--------CATWCT<br>MKNATTTGCATAYY	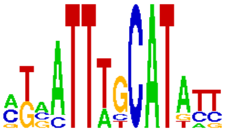
TAXCREB_02	3.9547e-02	---AGWATG------<br>GGGGRTATGCGTCAY	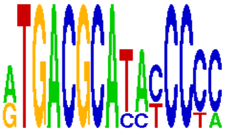
POU6F1_01	4.0839e-02	----AGWATG-<br>ATAAWTTATGC	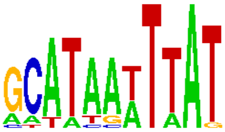

ZNF274W13

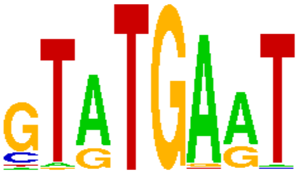

Motif Name	E-Value	Alignment	Weblogo
POU1F1_Q6	4.1655e-03	----ATTCATAC<br>WWTTATNCAT--	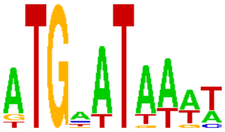

T	6.7534e-03	----GTATGAAT<br>CTAGGTGTGAA-	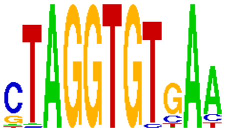
FOXP3_Q4	7.4820e-03	GTATGAAT---------<br>NTMTGNNANAACNNNWY	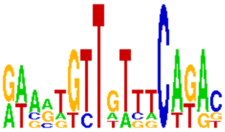
CAT8_Q6	1.2059e-02	--ATTCATAC<br>CCATTNNNCC	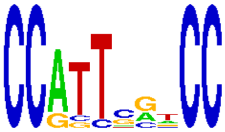
TBP_Q6	1.2913e-02	GTATGAAT-<br>ATATAAANR	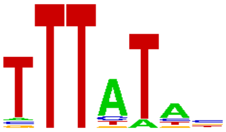

ZNF274W14

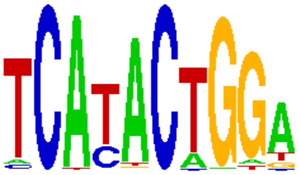

Motif Name	E-Value	Alignment	Weblogo
GRH_01	4.6281e-02	--TCCAGTATGA<br>AAACCRGT----	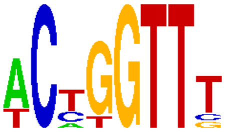
TAF_Q6	4.8037e-02	---TCATACTGGA--<br>TNGTCRTNTYNNACG	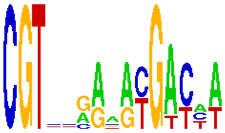
Pax6	5.1870e-02	---TCCAGTATGA-<br>MANTSAWGCGTGAA	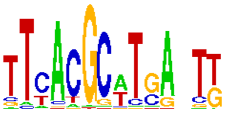
NKX25_01	5.5385e-02	TCATACTGGA<br>---CACTTRA	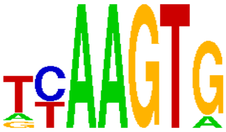

AR_01	6.2345e-02	--TCCAGTATGA---<br>GGWACANNNTGTNCT	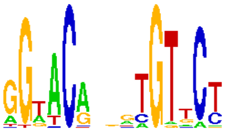

ZNF274W15 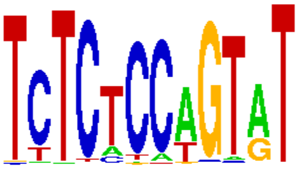

Motif Name	E-Value	Alignment	Weblogo
AREB6_01	1.4420e-02	ATACTGGAGAGA-<br>RNWCAGGTRNRNN	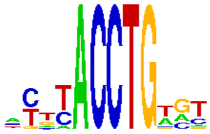
XPF1_Q6	5.2725e-02	TCTCTCCAGTAT<br>GKTSNYCNGA--	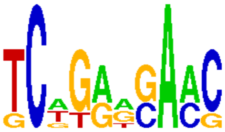
NKX25_01	7.6203e-02	TCTCTCCAGTAT<br>----TYAAGTG-	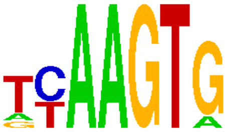
MAT1MC_02	8.4343e-02	TCTCTCCAGTAT--<br>----YCNATTGTYN	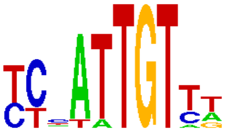
MAZ_Q6	8.4521e-02	TCTCTCCAGTAT<br>-CCCTCCCC---	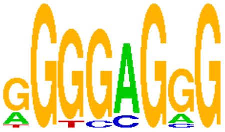

ZNF274W16 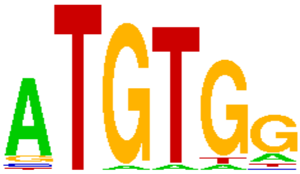

Motif Name	E-Value	Alignment	Weblogo

COREBINDINGFACTOR_Q6	2.6401e-03	---CCACAT<br>WNACCACA-	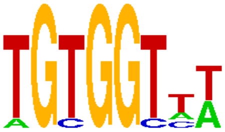
OSF2_Q6	2.8252e-03	-ATGTGG-<br>KNTGTGGT	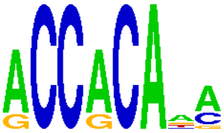
AML1_Q6	3.9747e-03	-CCACAT<br>ACCACA-	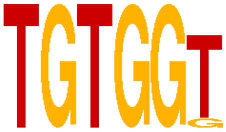
MIG1_01	4.6567e-03	----CCACAT-------<br>NNCCCCRSNTTTWWNNM	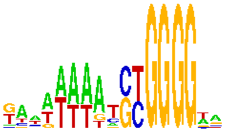
AML1_01	4.7720e-03	-CCACAT<br>ACCACA-	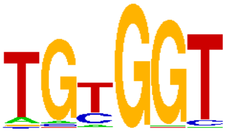

ZNF274W17

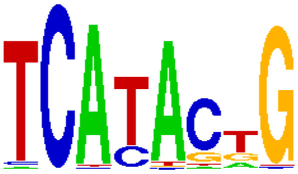

Motif Name	E-Value	Alignment	Weblogo
ZTA_Q2	1.6367e-02	----TCATACKG-<br>TGWGYCANNNTNW	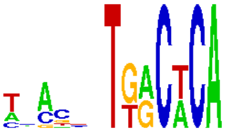
PAX3_01	3.5174e-02	--CMGTATGA---<br>KNAAGYGTGACGA	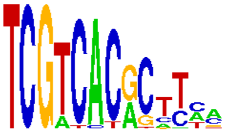
T	4.7132e-02	-TCATACKG--<br>TTCACACCTAG	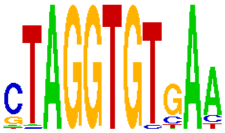

TCF11-MafG	4.7707e-02	-TCATACKG<br>GTCATN---	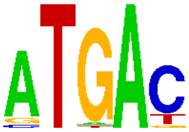
ICSBP_Q6	5.6168e-02	CMGTATGA----<br>CAGTTTCAYTTY	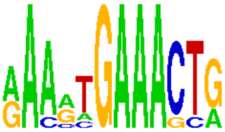

ZNF274W18

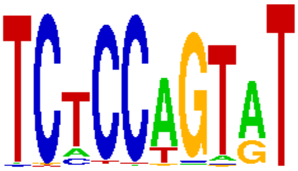

Motif Name	E-Value	Alignment	Weblogo
AREB6_01	2.9325e-02	---TCTCCAGTAT<br>NNYNYACCTGWNY	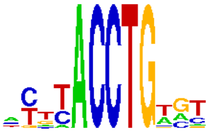
LXR_DR4_Q3	3.6831e-02	---TCTCCAGTAT---<br>TGACCGNNAGTAACCC	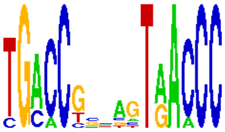
DR4_Q2	4.0065e-02	---TCTCCAGTAT----<br>TGNCCTNNNNTRACCYN	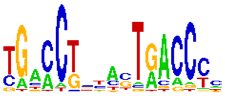
ZID_01	4.0541e-02	--TCTCCAGTAT-<br>NGGCTCYATCAYC	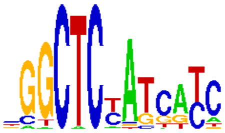
GRH_01	4.6988e-02	TCTCCAGTAT<br>AAACCRGT--	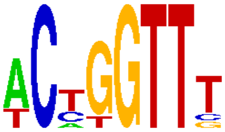

ZNF274W19

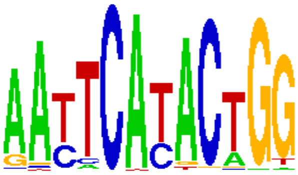

Motif Name	E-Value	Alignment	Weblogo
TBP_01	2.0814e-02	AATTCATACTGG<br>WATTTATA----	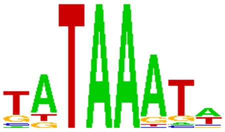
POU1F1_Q6	3.5658e-02	CCAGTATGAATT---<br>-----ATGNATAAWW	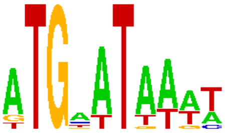
T	4.0952e-02	-CCAGTATGAATT<br>CTAGGTGTGAA--	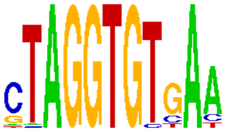
ISRE_01	5.1665e-02	CCAGTATGAATT----<br>-CAGTTTCWNTTTCNC	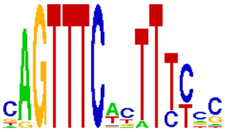
Prrx2	5.6801e-02	CCAGTATGAATT<br>-------TAATT	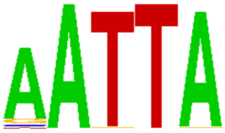

ZNF274W2

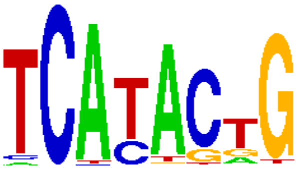

Motif Name	E-Value	Alignment	Weblogo
ZTA_Q2	1.5101e-02	----TCATACTG-<br>TGWGYCANNNTNW	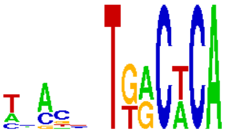

PAX3_01	2.4174e-02	--CAGTATGA---<br>KNAAGYGTGACGA	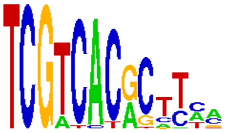
T	3.8818e-02	-TCATACTG--<br>TTCACACCTAG	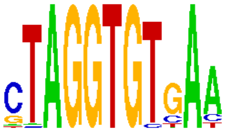
TCF11-MafG	5.0987e-02	-TCATACTG<br>GTCATN---	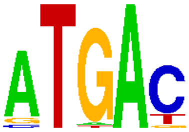
AP2REP_01	5.1036e-02	TCATACTG<br>-NCCACTG	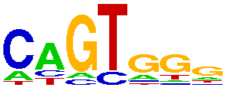

ZNF274W3 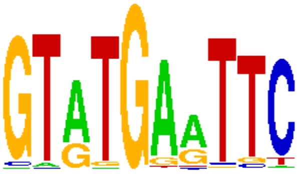

Motif Name	E-Value	Alignment	Weblogo
TBP_01	9.3221e-03	GAATTCATAC<br>-WATTTATA-	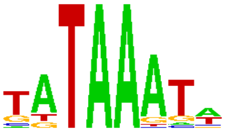
T	1.5946e-02	----GTATGAATTC<br>CTAGGTGTGAA---	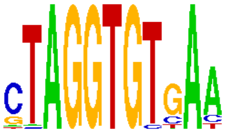
PAX_Q6	1.9267e-02	GTATGAATTC---<br>--GTKAGTTCCAG	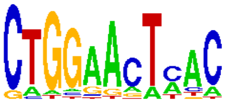
BRACH_01	2.3572e-02	GAATTCATAC--------------<br>AATTTCACACCTAGGTGTSANNNN	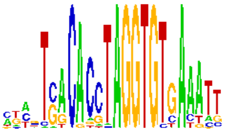

Pax6	2.8692e-02	-----GTATGAATTC<br>TTCACGCWTSANTK-	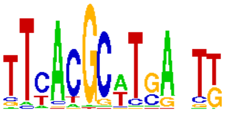

ZNF274W4 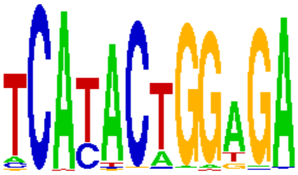

Motif Name	E-Value	Alignment	Weblogo
Pax6	3.6798e-02	-TCATACTGGAGA-<br>TTCACGCWTSANTK	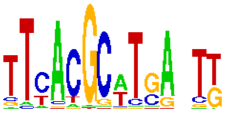
XPF1_Q6	6.4065e-02	--TCTCCAGTATGA<br>GKTSNYCNGA----	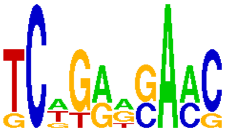
AREB6_01	7.1872e-02	---TCTCCAGTATGA<br>NNYNYACCTGWNY--	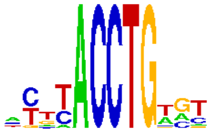
GRH_01	7.8203e-02	TCTCCAGTATGA<br>AAACCRGT----	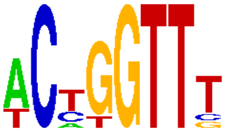
MAT1MC_02	8.5648e-02	TCTCCAGTATGA<br>--YCNATTGTYN	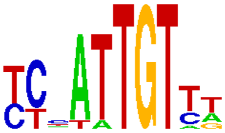

ZNF274W5 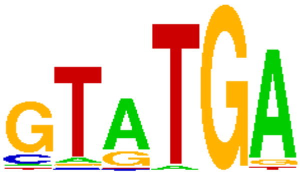

Motif Name	E-Value	Alignment	Weblogo

T	6.8688e-03	----GTATGA-<br>CTAGGTGTGAA	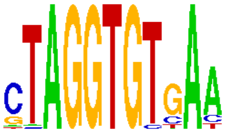
BRACH_01	9.2364e-03	--------------GTATGA----<br>NNNNTSACACCTAGGTGTGAAATT	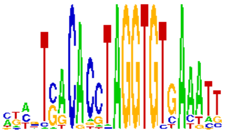
FOXP3_Q4	1.0621e-02	GTATGA-----------<br>NTMTGNNANAACNNNWY	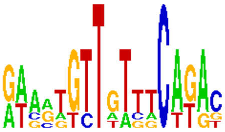
TAXCREB_02	1.4998e-02	----GTATGA-----<br>GGGGRTATGCGTCAY	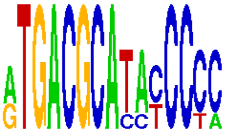
O2_01	2.3881e-02	----------GTATGA<br>YATCTACGTGGAATGA	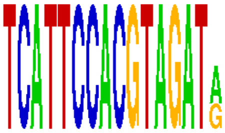

ZNF274W6

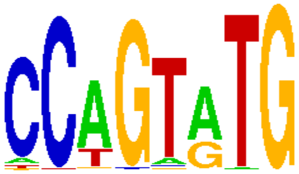

Motif Name	E-Value	Alignment	Weblogo
HAC1_Q2	3.0243e-02	--CATACTGG--<br>GACACGCTGKCM	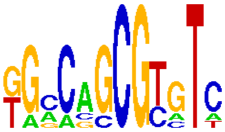
AREB6_01	4.2397e-02	CATACTGG------<br>-RNWCAGGTRNRNN	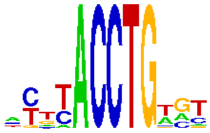
SOX9	4.2695e-02	CCAGTATG-<br>CYATTGTTN	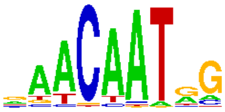

AP2REP_01	4.3978e-02	CATACTGG<br>NCCACTG-	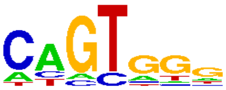
LXR_DR4_Q3	4.6573e-02	--CATACTGG------<br>GGGTTACTNNCGGTCA	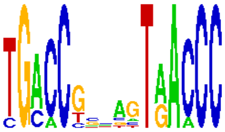

ZNF274W7

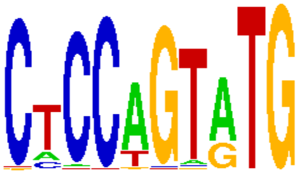

Motif Name	E-Value	Alignment	Weblogo
LXR_DR4_Q3	3.8787e-02	--CATACTGGAG----<br>GGGTTACTNNCGGTCA	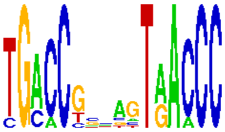
ZID_01	3.9355e-02	---CTCCAGTATG<br>NGGCTCYATCAYC	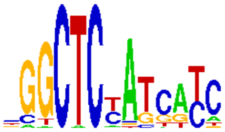
DR4_Q2	4.3031e-02	----CTCCAGTATG---<br>TGNCCTNNNNTRACCYN	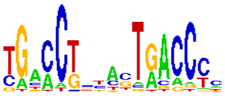
GRH_01	4.7514e-02	-CTCCAGTATG<br>AAACCRGT---	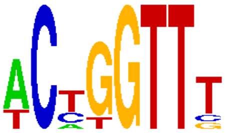
LXR_Q3	5.2235e-02	----CTCCAGTATG----<br>TGACCNNNAGTRACCCCA	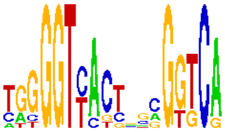

ZNF274W8

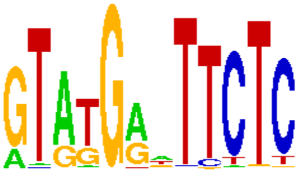

Motif Name	E-Value	Alignment	Weblogo
POU6F1_01	1.8029e-02	GTATGRNTTCTC<br>GCATAAWTTAT-	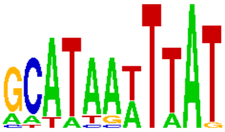
HSF_01	3.5346e-02	GTATGRNTTCTC<br>------NTTCT-	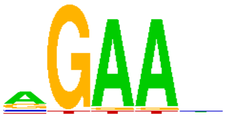
DL_02	4.5302e-02	GTATGRNTTCTC---<br>----NGNTTTTCYCN	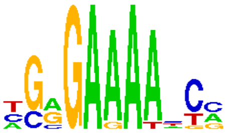
NFKAPPAB_01	4.6208e-02	GTATGRNTTCTC<br>--GGRAAKTCCC	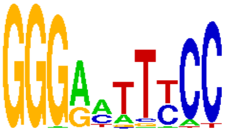
HSF_01	4.6462e-02	GAGAANYCATAC<br>-NGAAN------	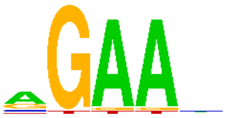

ZNF274W9

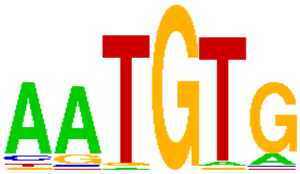

Motif Name	E-Value	Alignment	Weblogo
ZEN_Q6	7.1576e-03	---AATGTG<br>KTWAATGW-	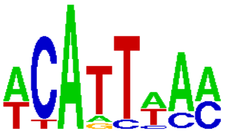

MIG1_01	7.9943e-03	-----CACATT------<br>NNCCCCRSNTTTWWNNM	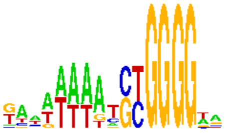
AP3_Q6	1.2612e-02	AATGTG--<br>AATTKRGA	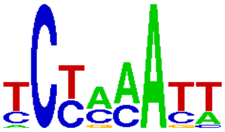
ALPHACP1_01	1.4962e-02	CACATT-----<br>CTCATTGGCTG	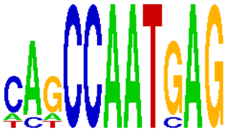
TEAD	1.6881e-02	CACATT------<br>YACATTCCWSNG	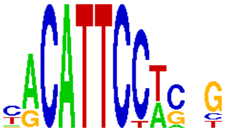

ZNF274F1

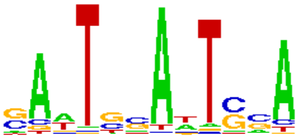

Motif Name	E-Value	Alignment	Weblogo
Staf	4.1550e-02	TNSANTNNANTS---------<br>-KCNNKGMANNMTGGGANNNN	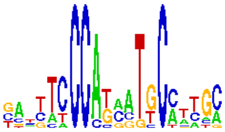
CHOP_01	5.3604e-02	-TNSANTNNANTS<br>GGSNATTGCAYNN	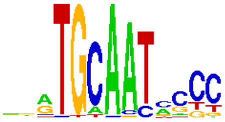
Chop-cEBP	5.9326e-02	TNSANTNNANTS<br>GSNATTGCAYNN	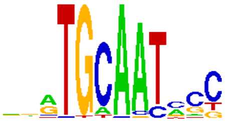
TEF1_Q6	9.0949e-02	SANTNNANTSNA<br>-----CATYYC-	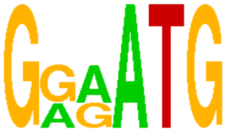

MADSB_Q2	1.0622e-01	TNSANTNNANTS------<br>---TTTSCATTTTTRGNW	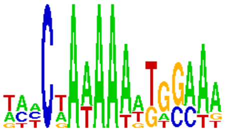

ZNF274F11

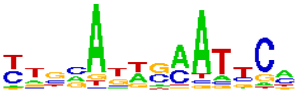

Motif Name	E-Value	Alignment	Weblogo
FTZ_01	5.3836e-02	NGNATKSNNTNNNR<br>--NNNNCAATTAAG	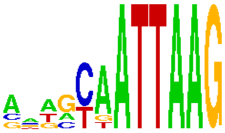
FOXM1_01	7.9620e-02	YNNNANNSMATNCN<br>----ANTCNANYT-	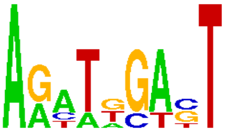
Prrx2	8.0524e-02	YNNNANNSMATNCN<br>-------TAATT--	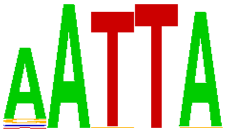
OCT1_Q5_01	1.1006e-01	NGNATKSNNTNNNR----<br>-------NATGNAAATNN	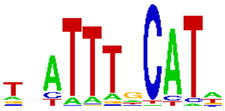
NKX25_02	1.1316e-01	YNNNANNSMATNCN<br>CWTAATTN------	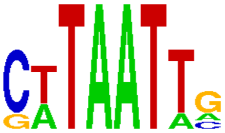

ZNF274M1

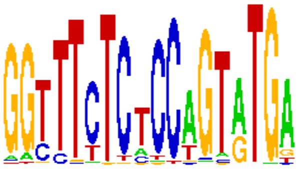

Motif Name	E-Value	Alignment	Weblogo

HSF_01	7.0517e-03	GGTTTCTCTCCAGTATGA<br>--NTTCT-----------	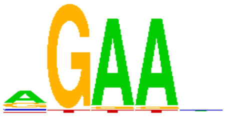
Pax5	1.6356e-02	-GGTTTCTCTCCAGTATGA-<br>YSGYYMCGCWNCRNNNNNCN	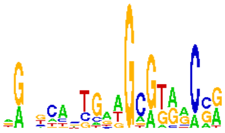
IRF1	4.0482e-02	TCATACTGGAGAGAAACC<br>------NAAANYGAAACC	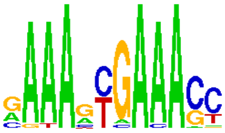
SPIB	4.2475e-02	GGTTTCTCTCCAGTATGA<br>--TTCCKST---------	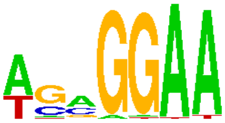
DL_02	4.2910e-02	GGTTTCTCTCCAGTATGA<br>NGNTTTTCYCN-------	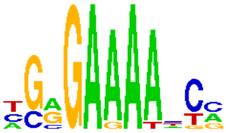

ZNF274M11

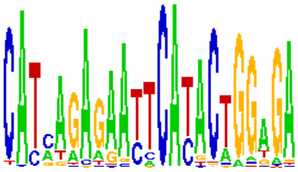

Motif Name	E-Value	Alignment	Weblogo
GAL4_01	6.7319e-07	CATMAGAGAATTCATACTGGAGA<br>STTCGGASNANWGTNNNCCGNNN	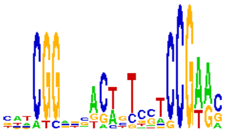
IRF1_Q6	5.5133e-03	CATMAGAGAATTCATACTGGAGA<br>---AANTGAA-------------	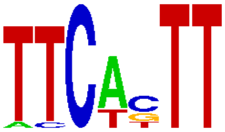
LEF1_Q2	6.9736e-03	TCTCCAGTATGAATTCTCTKATG<br>---------------CTTTGA--	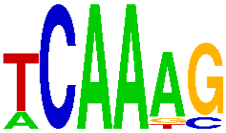

DR3_Q4	1.2251e-02	-----CATMAGAGAATTCATACTGGAGA<br>ANNNGGKTCANNARGKNCAYY-------	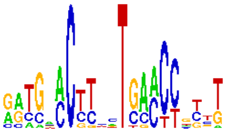
ISRE_01	1.7716e-02	TCTCCAGTATGAATTCTCTKATG<br>----CAGTTTCWNTTTCNC----	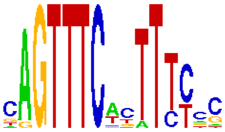

ZNF274M12

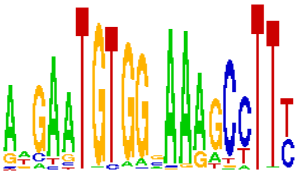

Motif Name	E-Value	Alignment	Weblogo
MNB1A	2.2725e-03	RAAGGCTTTNCCACATTCNT<br>----NCTTT-----------	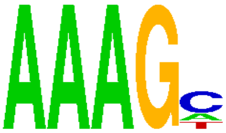
PBF	2.7190e-03	RAAGGCTTTNCCACATTCNT<br>----RCTTT-----------	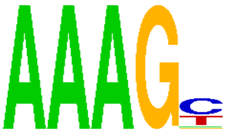
E2F1DP1_01	4.9972e-03	RAAGGCTTTNCCACATTCNT<br>------TTTCSCGC------	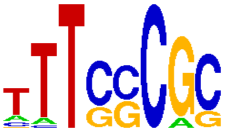
E2F1DP2_01	5.1529e-03	ANGAATGTGGNAAAGCCTTY<br>------GCGGGAAA------	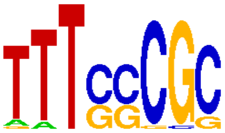
DL_02	9.8809e-03	ANGAATGTGGNAAAGCCTTY<br>-------NGRGAAAANCN--	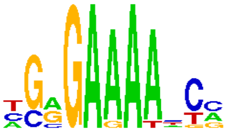

ZNF274M13

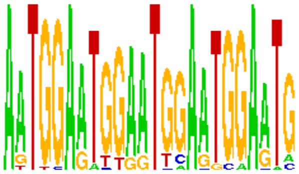

Motif Name	E-Value	Alignment	Weblogo
TEF1_Q6	5.4332e-04	CATTCCATTCCATTCCATTCCATT<br>----------CATYYC--------	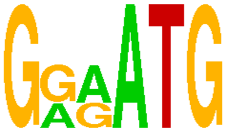
HSF_02	6.0892e-02	CATTCCATTCCATTCCATTCCATT<br>-NTTCNNTTCNNTTCN--------	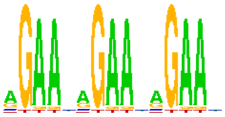
HSF_02	9.3615e-02	CATTCCATTCCATTCCATTCCATT<br>-NTTCTNTTCTNTTCT--------	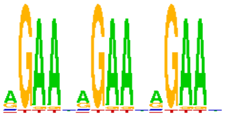
CAT8_Q6	1.1105e-01	AATGGAATGGAATGGAATGGAATG<br>----------GGNNNAATGG----	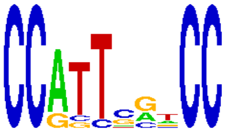
DTYPEPA_B	1.3120e-01	AATGGAATGGAATGGAATGGAATG<br>------GTTTAATGGT--------	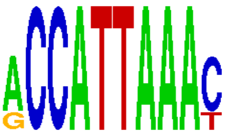

ZNF274M2

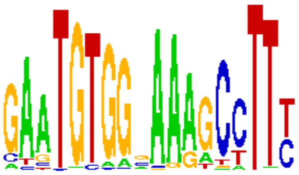

Motif Name	E-Value	Alignment	Weblogo
MNB1A	3.8399e-03	RAAGGCTTTNCCACATTC<br>----NCTTT---------	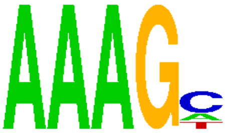

PBF	4.4981e-03	RAAGGCTTTNCCACATTC<br>----RCTTT---------	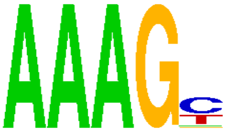
E2F1DP1_01	7.1875e-03	RAAGGCTTTNCCACATTC<br>------TTTCSCGC----	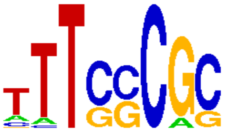
E2F1DP2_01	7.3104e-03	GAATGTGGNAAAGCCTTY<br>----GCGGGAAA------	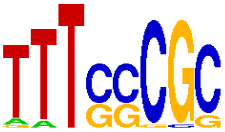
E2F4DP2_01	1.5221e-02	GAATGTGGNAAAGCCTTY<br>----GCGSGAAA------	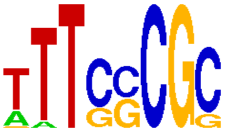

ZNF274M3

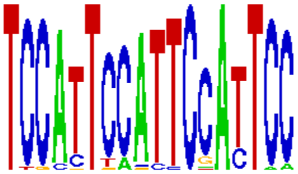

Motif Name	E-Value	Alignment	Weblogo
TEF1_Q6	3.9713e-03	TCCATTCCATTCCATTCC<br>--CATYYC----------	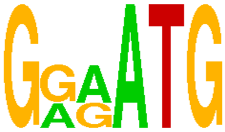
HSF_02	2.1646e-02	GGAATGGAATGGAATGGA<br>NGAANNGAANNGAAN---	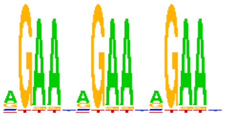
HSF_02	3.8808e-02	TCCATTCCATTCCATTCC<br>---NTTCTNTTCTNTTCT	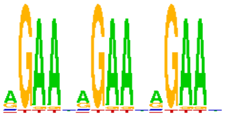
CAT8_Q6	1.0045e-01	TCCATTCCATTCCATTCC<br>------CCATTNNNCC--	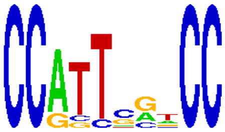

TEAD	1.0853e-01	GGAATGGAATGGAATGGA<br>-CNSWGGAATGTR-----	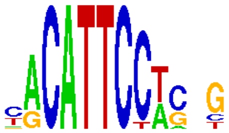
