# Supplementary material for: ZNF274 Recruits the Histone Methyltransferase SETDB1 to the 3′ Ends of ZNF Genes
Source: PLoS One. 2010 Dec 8;5(12):e15082. doi: 10.1371/journal.pone.0015082 (PMC2999557; doi:10.1371/journal.pone.0015082)
Supplement: Figure S6 — Comparison of peak sets identified using ChIP-chip and ChIP-seq for H3K9me3, SETDB1, and KAP1. An example of the binding patterns for H3K9me3, SETDB1, and KAP1 using the different platforms is shown in panel A. The ChIP-chip vs. ChIP-seq binding patterns for H3K9me3, for SETDB1, and KAP1 are very similar. However, the ChIP-chip data has considerably more background and the data is compressed into a smaller range (such that it is difficult to distinguish the very highest peaks from the moderately high peaks). For H3K9me3, we identified 1497 ChIP-chip peaks and 3866 ChIP-seq peaks on chromosome 19, and found that half of the H3K9me3 peaks from obtained using one platform are represented in the dataset obtained using the other platform (panel B); in general, these common peaks are found in regions displaying the highest and widest coverage by H3K9me3. The peaks that did not overlap were narrower peaks that tended to be difficult to analyze in one of the experimental platforms. For example, some regions were not represented on the array because of the spacing of the oligonucleotides and some regions were not represented in the sequenced tags because they matched to more than one place on the genome. We next compared the SETDB1 and KAP1 peak sets obtained using ChIP-chip vs. ChIP-seq. Again, the ChIP-seq data was much more robust, having higher signal to noise than the ChIP-chip data. Most of the SETB1 (83%) and KAP1 (95%) ChIP-seq peaks were contained within the ChIP-chip peak sets, suggesting that the extra peaks in the ChIP-chip sets are false positives. (PDF) [file pone.0015082.s006.pdf]

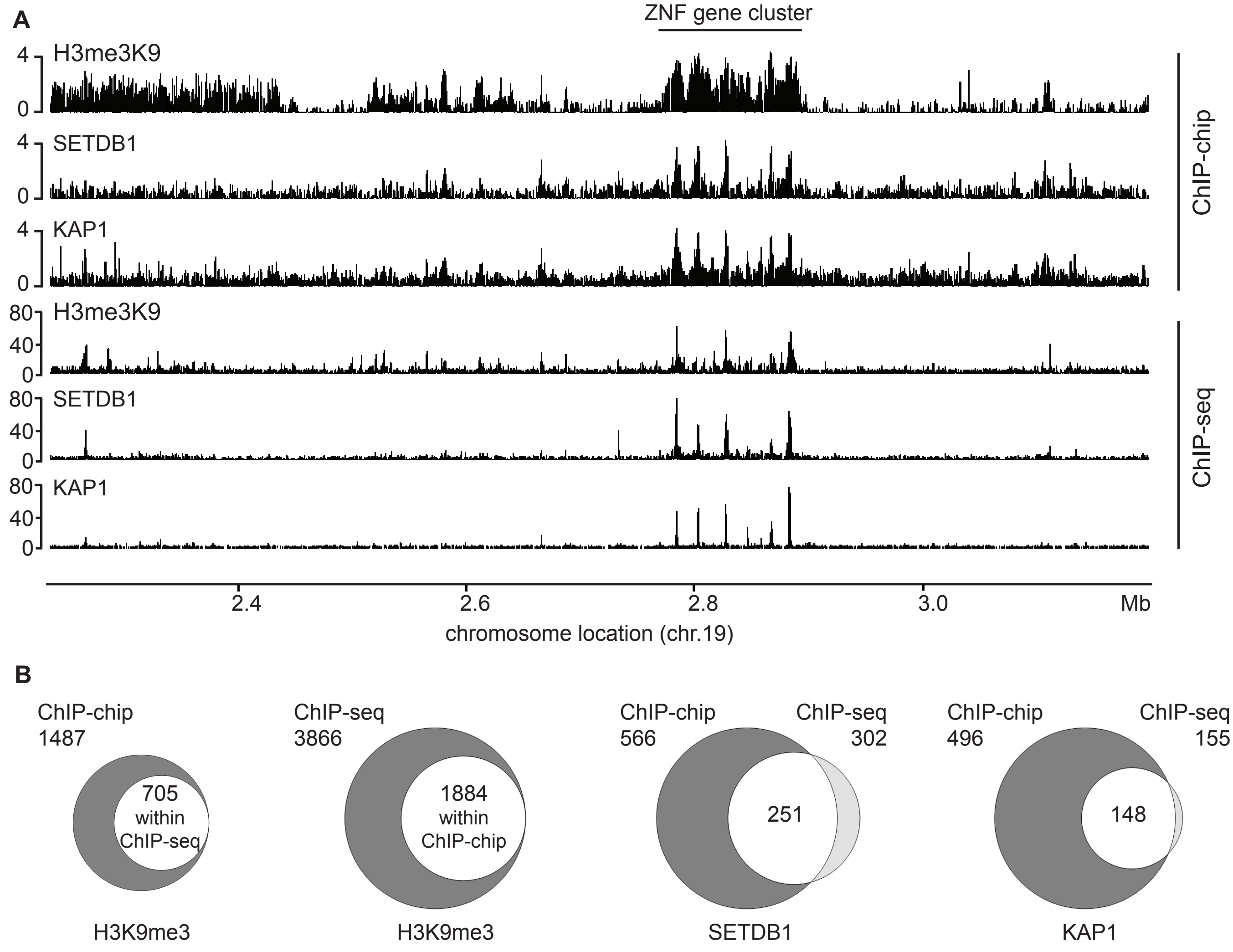

Frietze_Figure S6. Comparison of peak sets identified using ChIP-chip and ChIP-seq for H3K9me3, SETDB1, and KAP1. An example of the binding patterns for H3K9me3, SETDB1, and KAP1 using the different platforms is shown in panel A. The ChIP-chip vs. ChIP-seq binding patterns for H3K9me3, for SETDB1, and KAP1 are very similar. However, the ChIP-chip data has considerably more background and the data is compressed into a smaller range (such that it is difficult to distinguish the very highest peaks from the moderately high peaks). For H3K9me3, we identified 1497 ChIP-chip peaks and 3866 ChIP-seq peaks on chromosome 19, and found that half of the H3K9me3 peaks from obtained using one platform are represented in the dataset obtained using the other platform (panel B); in general, these common peaks are found in regions displaying the highest and widest coverage by H3K9me3. The peaks that did not overlap were narrower peaks that tended to be difficult to analyze in one of the experimental platforms. For example, some regions were not represented on the array because of the spacing of the oligonucleotides and some regions were not represented in the sequenced tags because they matched to more than one place on the genome. We next compared the SETDB1 and KAP1 peak sets obtained using ChIP-chip vs. ChIP-seq. Again, the ChIP-seq data was much more robust, having higher signal to noise than the ChIP-chip data. Most of the SETB1 (83%) and KAP1 (95%) ChIP-seq peaks were contained within the ChIP-chip peak sets, suggesting that the extra peaks in the ChIP-chip sets are false positives.
