# Supplementary material for: ZNF274 Recruits the Histone Methyltransferase SETDB1 to the 3′ Ends of ZNF Genes
Source: PLoS One. 2010 Dec 8;5(12):e15082. doi: 10.1371/journal.pone.0015082 (PMC2999557; doi:10.1371/journal.pone.0015082)
Supplement: Figure S7 — Heatmap of expression data for ZNF274 targets. The expression levels of the subset of ZNF274 target genes (identified as the nearest gene to each binding site from the K562 ZNF274 ChIP-seq dataset) present on the Affymetrix Human Exon 1.0 ST (HuEx-1_0-st-v2) Arrays is shown compared to the expression levels of the same number of genes from each of the 5 quintiles representing expression of all mRNAs in K562 cells. (PDF) [file pone.0015082.s007.pdf]

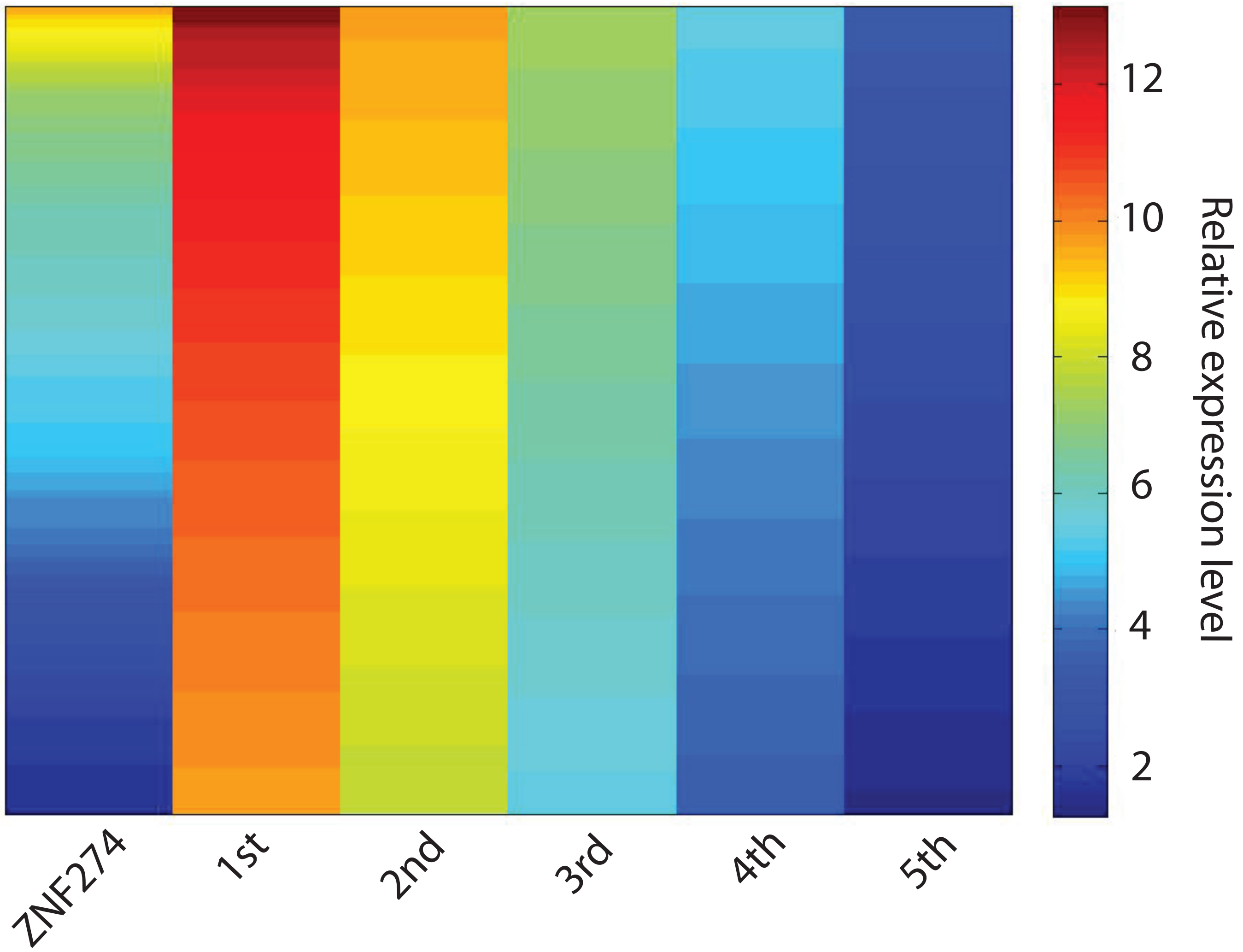

Frietze_Figure S7. Heatmap of expression data for ZNF274 targets. The expression levels of the subset of ZNF274 target genes (identified as the nearest gene to each binding site from the K562 ZNF274 ChIP-seq dataset) present on the Affymetrix Human Exon 1.0 ST (HuEx-1_0-st-v2) Arrays is shown compared to the expression levels of the same number of genes from each of the 5 quintiles representing expression of all mRNAs in K562 cells.
