# Supplementary figures and images for: ZNF274 Recruits the Histone Methyltransferase SETDB1 to the 3′ Ends of ZNF Genes
Source: PLoS One. 2010 Dec 8;5(12):e15082. doi: 10.1371/journal.pone.0015082 (PMC2999557; doi:10.1371/journal.pone.0015082)

H3K9me3

ZNF274

KAP1

SETDB1

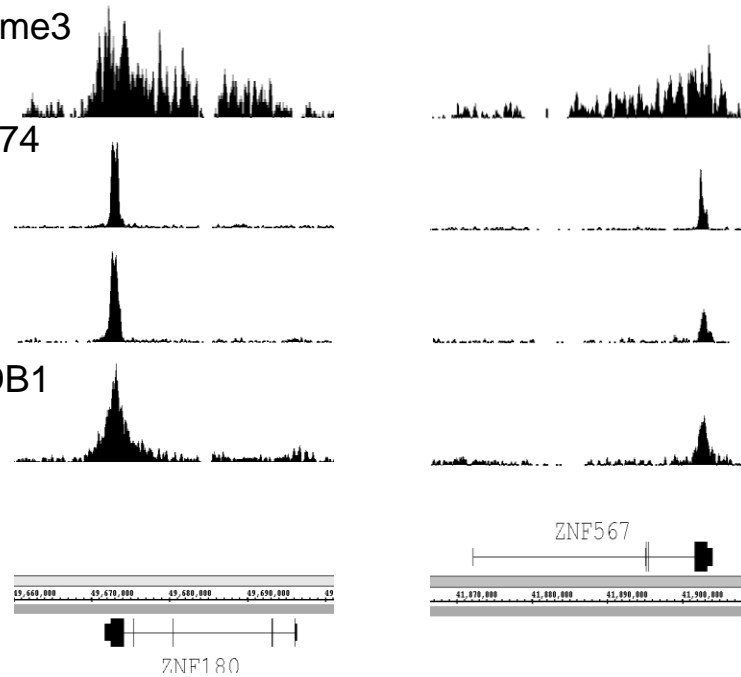

H3K9me3

ZNF274

KAP1

SETDB1

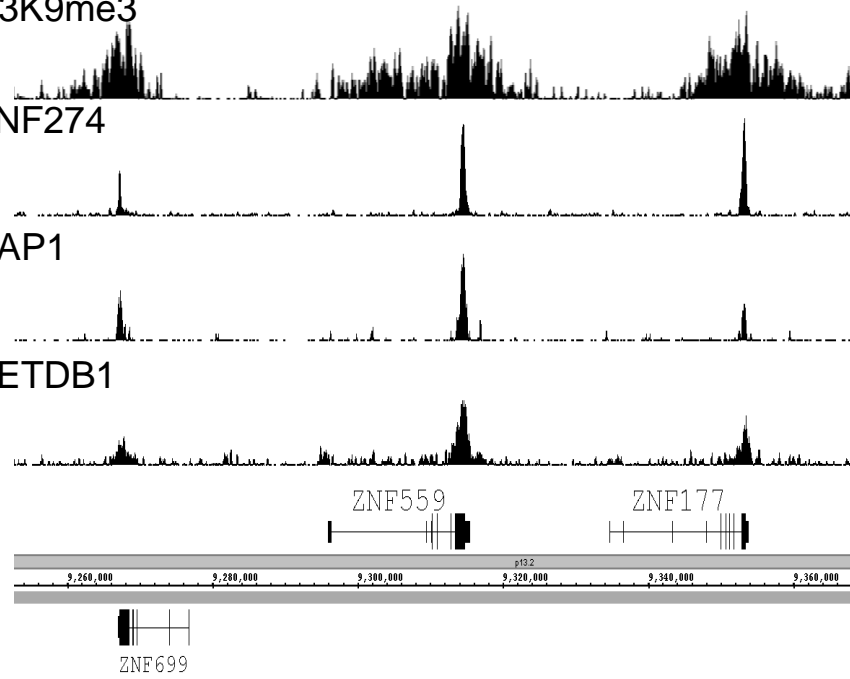

Supplement: Figure S9 — ChIP-seq binding patterns of ZNF274, KAP1, SETDB1, and H3K9me3 at individual gene loci. Shown are snapshots of the ChIP-seq data at specific C2H2 ZNF genes. (PDF) [file pone.0015082.s009.pdf]
