# Supplementary material for: ZNF274 Recruits the Histone Methyltransferase SETDB1 to the 3′ Ends of ZNF Genes
Source: PLoS One. 2010 Dec 8;5(12):e15082. doi: 10.1371/journal.pone.0015082 (PMC2999557; doi:10.1371/journal.pone.0015082)
Supplement: Figure S10 — Shown is a comparison of ZNF274 binding sites, the location of all C2H2 ZNF genes, and the location of the identified 29mer motif (with 2 allowed mismatches) in forward and reverse orientations across the right arm of chromosome 19. There is a high correspondence between ZNF274 binding sites and the subset of C2H2 ZNF genes that contain the 29mer motif. (PDF) [file pone.0015082.s010.pdf]

ZNF274 ChIP-seq data for Chr19 q12-q13.43

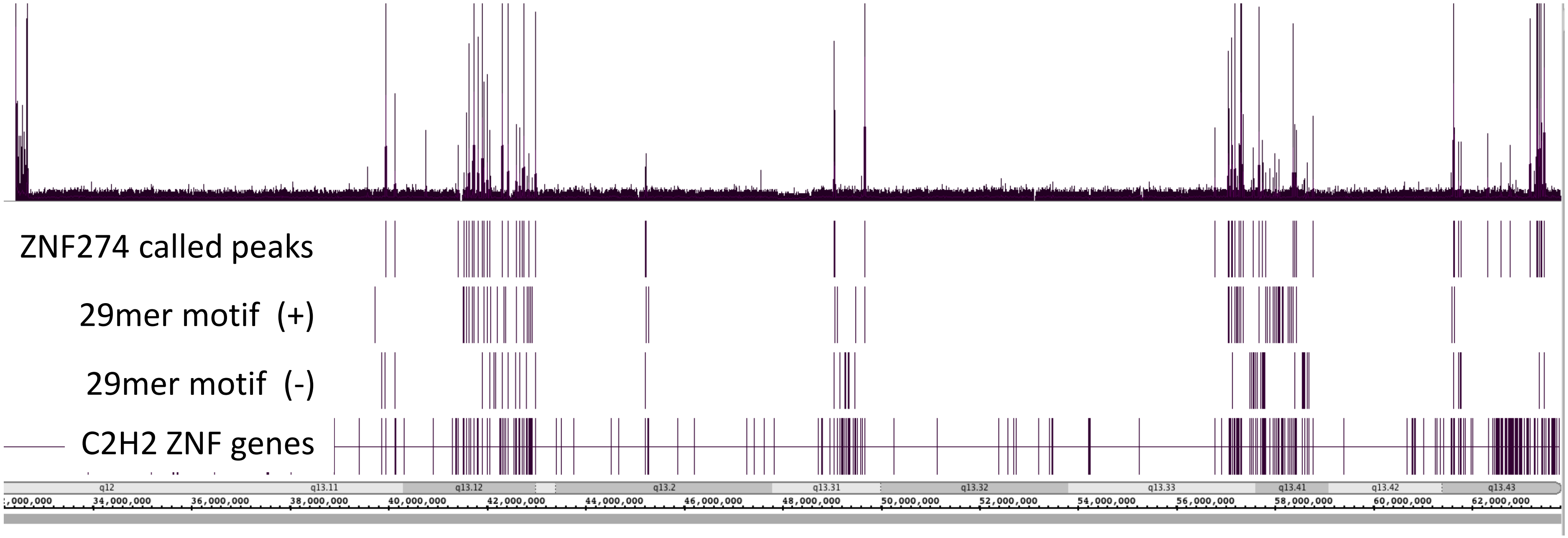

Figure S10. Shown is a comparison of ZNF274 binding sites, the location of all C2H2 ZNF genes, and the location of the identified 29mer motif (with 2 allowed mismatches) in forward and reverse orientations across the right arm of chromosome 19. There is a high correspondence between ZNF274 binding sites and the subset of C2H2 ZNF genes that contain the 29mer motif.
