# Supplementary material for: ZNF274 Recruits the Histone Methyltransferase SETDB1 to the 3′ Ends of ZNF Genes
Source: PLoS One. 2010 Dec 8;5(12):e15082. doi: 10.1371/journal.pone.0015082 (PMC2999557; doi:10.1371/journal.pone.0015082)
Supplement: Figure S8 — Motifs identified inZNF274 binding sites. (A) Motifs identified within 50 nucleotides of either side of the center of ZNF274 binding sites. Also indicated is the number of occurrences of each motif in the 337 ZNF274 binding sites identified in K562 cells. Both ZNF274M11 and ZNF274M1 are present in 72 of the top 100 ranked ZNF274 binding sites from K562 cells. (B) The overlap of the 5′ end of the reverse orientation of the M1 motif and the 3′ end of the M11 motif. (C) The sequence of the full 29mer. (D) The reverse orientation of the 29mer encodes the conserved linker and the conserved H2 helix found in C2H2 zinc fingers. (PDF) [file pone.0015082.s008.pdf]

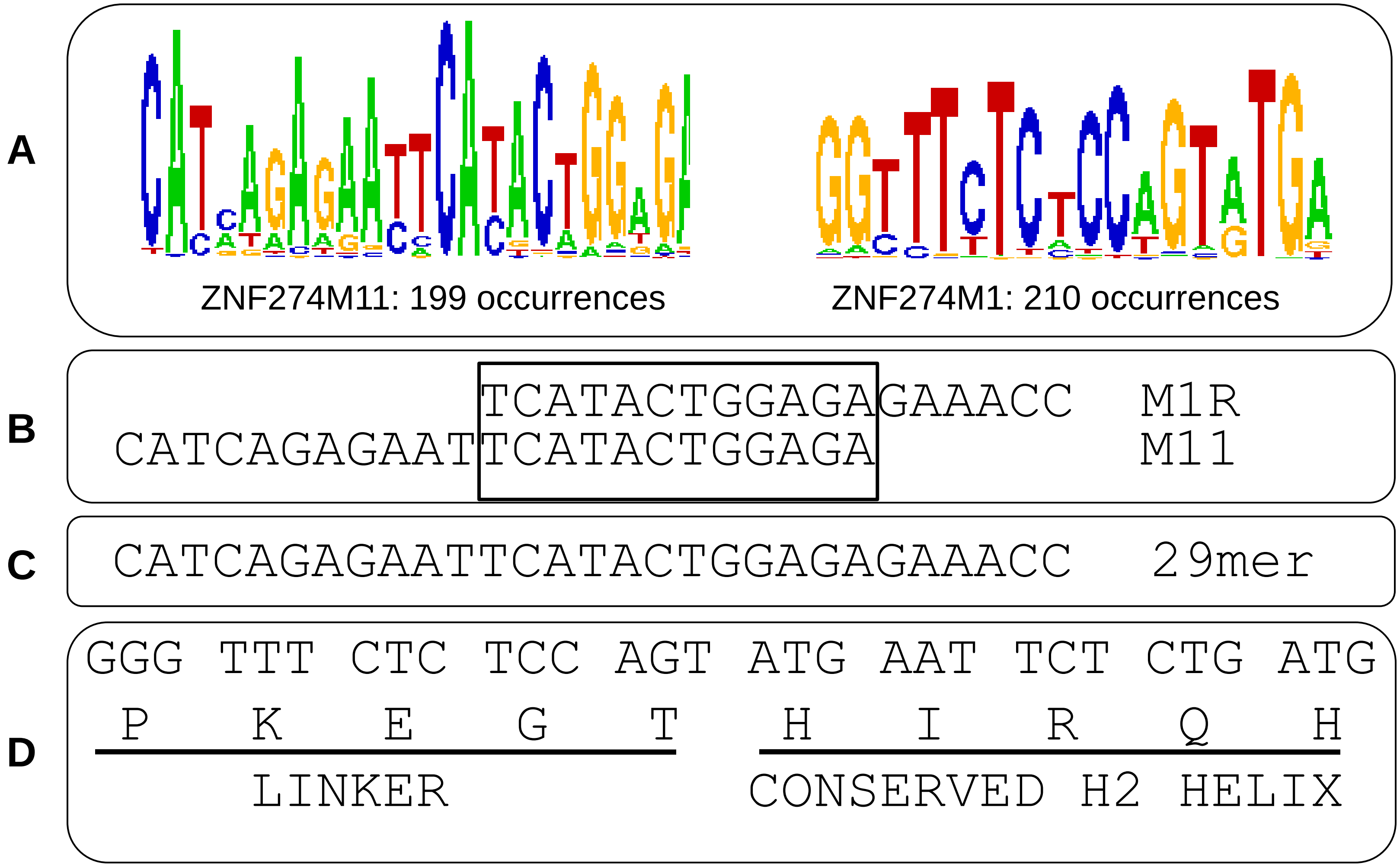

Frietze_Figure S8. Motifs identified in ZNF274 binding sites. (A) Motifs identified within 50 nucleotides of either side of the center of ZNF274 binding sites. Also indicated is the number of occurrences of each motif in the 337 ZNF274 binding sites identified in K562 cells. Both ZNF274M11 and ZNF274M1 are present in 72 of the top 100 ranked ZNF274 binding sites from K562 cells. **(B)** The overlap of the 5' end of the reverse orientation of the M1 motif and the 3' end of the M11 motif. **(C)** The sequence of the full 29 mer. **(D)** The reverse orientation of the 29 mer encodes the conserved linker and the conserved H2 helix found in C2H2 zinc fingers.
